# Efficient distribution of tuned mass dampers for seismic control in regular and irregular multi-story steel buildings

**DOI:** 10.1038/s41598-026-55777-2

**Published:** 2026-06-11

**Authors:** Mohamed M. Shabib, Hassan M. Farag, Yasser A. Khalifa, M. S. Zahran

**Affiliations:** Civil Engineering Department, Military Technical College, Cairo, Egypt

**Keywords:** TMD, seismic excitation, mass ratio, Passive control, MSB, Vibrations, Engineering, Mathematics and computing

## Abstract

Passive control systems, such as tuned mass dampers (TMDs), are widely used to reduce the lateral response of multi-story buildings (MSBs) without external power. This paper presents a numerical investigation of selected TMD distribution layouts in regular and irregular 12-story steel buildings under a limited set of scaled earthquake records. Although single-tuned mass damper (STMD) and multi-tuned mass damper (MTMD) systems have been widely studied, many previous investigations have focused mainly on tuning parameters, mass ratios, or simplified damper locations, with less attention to how an equivalent auxiliary mass should be spatially distributed to address coupled lateral–torsional response in asymmetric plans. Using ABAQUS/CAE for nonlinear time-history analysis, this study compares conventional STMDs with selected vertical, horizontal, and perimeter-based MTMD configurations. For the regular building, the horizontal four-corner MTMD layout was further examined through a detuning sensitivity analysis using frequency deviations of 0%, ± 2%, ± 4%, ± 6%, and ± 8%. Among the investigated detuning levels, the ± 4% case produced the lowest maximum top lateral displacement and inter-story relative displacement for the selected records. For irregular buildings (T, C, L, and stadium shapes), the study evaluates a dynamic-eccentricity-based MTMD placement strategy in which damper coordinates are selected based on CM–CR eccentricity and torsional lever arms. Under the selected scaled records, the perimeter-based MTMD arrangement reduced the maximum top-floor displacement by 18.3% − 52.3% and the maximum top-floor torsional rotation by 25.4%–68.0% across the investigated irregular configurations. The corresponding reductions in base shear and maximum top-floor acceleration ranged from 15.2% to 31.3% and from 12.2% to 34.7%, respectively. These findings are limited to the investigated structural models, selected damper configurations, engineering demand parameters, and scaled ground-motion records.

## Introduction

Active vibrations caused by seismic activity, explosions, and extreme winds create destructive dynamics, which are a significant issue for both structural and performance safety. Correspondingly, structural engineering has adapted various methods of control, i.e., passive, active, semi-active, and hybrid damping systems, as effective means to contain these dynamic responses^1–5^. Passive energy dissipation systems, such as base isolation, viscoelastic, friction, and TMDs^6–12^, have been developed and adopted at a very high rate. Since these systems require no external power, they offer significant benefits over active and semi-active controls, including reduced costs and more effective vibration suppression. TMDs are classified as conventional, pendular, bidirectional, and tuned liquid column dampers (TLCDs)^13^. Traditional TMD, which is a passive device with a mass, spring, and viscous damper, is used to mitigate structural vibrations.

Beyond conventional TMD and MTMD systems, recent studies have proposed advanced vibration-control devices to increase energy dissipation, broaden the effective frequency range, or reduce seismic excitation. Negative-stiffness-based amplifying dampers use a negative-stiffness mechanism to amplify the relative deformation transmitted to the damping element to increase the energy dissipation. However, their application requires explicit stability assessment and clearly defined design limits when nonlinear structural response is considered^14,15^. Inerter-based devices, such as tuned viscous mass dampers, tuned mass damper-inerter systems, and inerter-enabled isolators, can achieve the apparent mass-amplification effect without an equivalent physical mass but typically involve special mechanical components and device-level optimization, as well as specific assumptions about installation and force transfer^16,17^. Other recent developments, such as butterfly-shaped damping-integrated tuned viscous mass dampers and vertical inerter-based dampers, have also been proposed to improve the damping and acceleration control in particular structural applications^18,19^. Seismic isolation is also effective in reducing seismic demand. It may reduce torsional coupling by tuning the stiffness and planar distribution of isolation bearings such that the effective stiffness center approaches the center of mass^20^.

These methods are relevant alternatives to the present problem, but they require device configurations and implementation assumptions that differ from the floor- or roof-level auxiliary-mass strategy considered here. Therefore, MTMDs were selected in this study not as a universally superior control technology, but as a mechanically simple, fully passive strategy suitable for examining how the same total auxiliary mass can be divided into several units and positioned to influence lateral and torsional response in multi-story steel buildings.

Numerous studies have been made to improve seismic performance through the use of multiple tuned mass dampers, in which units are tuned to various structural modes and distributed throughout the structure. Research in this field has progressed from simple analytical studies to complex optimization and comparison studies. As an example, the seismic case analytical procedures examined the impacts of structural irregularities^21^. Meanwhile, Valmundsson and Nau (1997) investigated anomalies arising from variations in mass, strength, and stiffness in normal buildings^22^. Reddy et al. (2018) set the critical parameters of TMD efficacy in their study; they concluded that tuned mass dampers would best extinguish vibrations with a mass ratio of 5% and at the frequency of the structure^23^. The mass component itself was fundamental to the argument by Bagheri and Rahmani Dabbagh^24^, which determined that a viscous damper was not necessarily required to achieve an optimum mass damper. TMDs have been proven to be effective in different structures.

In the case of irregular buildings, Lin et al. (2017) had to develop a special tuned mass damper using three-degree-of-freedom modal properties^25^. One of the key concerns of the recent literature is the efficiency of the design and location of the MTMD. The dominance of distributed systems has been validated by Elias et al. (2019), who established the positive effects of the distribution of multiple mass vibration absorbers on earthquake-vibrated structures^26^. This was further examined by Rahmani and Konke (2019) through the use of genetic algorithms, and they found that building-height-distributed dampers are more efficient in higher modalities, and their optimal position depends on the frequency of the earthquake^27^. To simplify the optimization process, which is computationally intensive, Yucel et al. (2019) were able to use an artificial neural network (ANN) to predict TMD parameters^28^.

Additionally, as part of the optimization methods, Keshtegar and Etedali (2018) provided a technique that is based on the dynamic parameters of a structure to compute TMD more accurately^29^. Nigdeli and Bekdas (2019) enhanced the effectiveness of distributed placement by examining the analysis of the fact that dampers placed at multiple positions in the structure are more effective^30^. Hao et al. (2021) researched a Tuned Viscous Mass Damper (TVMD) in the case of nearby building structures, also created the best design approaches, and showed TVMD had better energy dissipation and response mitigation as compared to traditional dampers that had less mass and lower damping requirements^31^. Ocak et al. (2022) optimized five liquids in Tuned Liquid Dampers (TLDs) to control seismic code in buildings; the mass and height of the structure matter in terms of optimal liquid selection (density and viscosity) with the help of the Adaptive Harmony Search algorithm^32^.

Jia et al. (2023) explored the use of the Tuned Viscous Mass Damper (TVMD) as seismic control in core wall structures. When the system was switched to the second mode, much of the inter-story drift and floor acceleration was dramatically reduced by 23% and 36%, respectively, compared with standard viscous damper outriggers^33^. Kontoni and Farghaly (2023) investigated irregular steel high-rise buildings that have structural interaction with soil. They concluded that placing TMDs at the top corners and at the upper half of the building’s height are the best mitigation options for seismic response^34^. Fadaie et al. (2025) propose the usage of a hybrid solution that employs vertical mass isolation (VMI) and tuned mass dampers (TMDs). This type of structure causes a significant decrease in the seismic and harmonic response and a maximum 25% and 35% decrease in the displacement and shear on the base, respectively, compared with the traditional structures, and an excellent level of structural performance^35^.

Recent work has further shifted attention from single-device tuning toward the distribution of several TMD or MTMD units over the height of the structure. Ozturk et al. optimized the vertical location and design of multiple TMDs under seismic excitation and showed that the floor level at which the auxiliary masses are installed has a direct effect on the response-reduction efficiency^36^. Akyurek compared different height-wise MTMD placement strategies and emphasized that the effectiveness of MTMDs depends on both the targeted modes and the selected floor locations^37^. Similarly, Akhlagh Pasand and Zahrai investigated vertically distributed MTMDs in tall buildings and reported that using more than one mode for tuning and distributing dampers can help address higher-mode effects that are difficult to control using a conventional STMD^38^. Naderpour et al. also evaluated TMD placement at different building levels, supporting the broader observation that damper location is an important design variable rather than a secondary modeling assumption^39^.

More recent optimization-based studies have also considered the distribution of MTMDs from a design-efficiency perspective. Chan proposed an inverse element exchange method for optimizing MTMD distribution and compared it with STMD, uniformly distributed MTMDs, and genetic-algorithm-based layouts. This study showed that optimized distributions can improve inter-story drift response while reducing the computational effort associated with conventional search procedures^40^. Djerouni et al. investigated MTMD schemes for mitigating seismic pounding in adjacent buildings and compared top-level MTMDs with floor-distributed MTMDs, further confirming that the distribution pattern can influence the control performance^41^. Reliability- and robustness-oriented studies have also considered optimum MTMD design under earthquake excitation, including probabilistic and random-excitation frameworks^42^.

Despite these advances, most recent distributed-MTMD studies have mainly focused on vertical floor placement, parameter optimization, inter-story drift reduction, pounding mitigation, or reliability-based design. Comparatively less attention has been given to plan-wise MTMD distribution in asymmetric buildings, where the damper coordinates need to be related to the center of mass (CM), center of rigidity (CR), structural eccentricity, and torsional lever arms. This point is particularly relevant to irregular buildings because their seismic response is governed not only by lateral translation but also by coupled lateral-torsional motion.

To address this gap, the present study first compares STMD, vertically distributed MTMDs, and horizontally distributed MTMDs in a regular 12-story steel building using the same total auxiliary mass. The horizontal four-corner MTMD layout is then examined through a detuning sensitivity analysis using frequency deviations of 0%, ± 2%, ± 4%, ± 6%, and ± 8%, with the response evaluated using top displacement, inter-story relative displacement, floor acceleration, base shear, overturning moment, damper stroke, and damper force. This additional comparison is included to clarify the influence of detuning on the selected horizontal MTMD layout, rather than to propose a universally optimized detuning bandwidth. The selected distribution concept is then extended to T-, C-, L-, and stadium-shaped steel buildings. The main contribution is the numerical evaluation of a CM-CR/eccentricity-based MTMD placement strategy in which damper coordinates are selected according to their torsional lever arms relative to the CR, rather than by using only roof-centered or uniformly distributed arrangements.

## FEA setup of the proposed models

### Numerical setting of the models

**Fig. 1:**
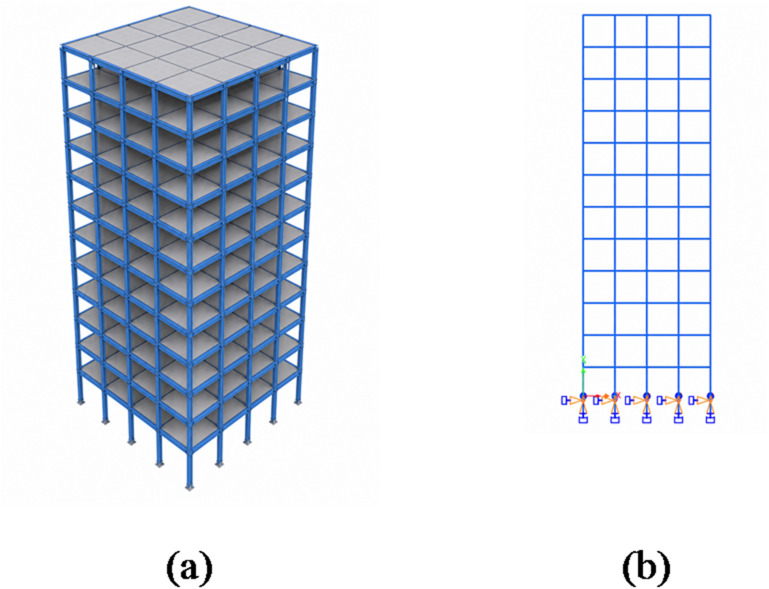
(a) 3D steel model, (b) finite element constraint.

Tables 1 and 2, and 3 display the load data, material properties^43^, and the model specification, respectively. The finite element method was used in this research to construct the 3D proposed steel model^44,45^, showing a 3D model, and finite element constraint as fixed support conditions, respectively, as shown in Fig. 1.

**Table 1:** Load data.

Load	According to ECP 201:2012
Floor covering load	1.5 kN/m^2^
Live load	2 kN/m^2^
Wall load	7 kN/m

The Egyptian Code for Load Calculations stipulates that design loads are utilized to ascertain the self-weight of structural elements and the loads from floor coverings. Specifically, it corresponds to a part of the live load in addition to the total permanent load. For residential buildings, this percentage is calculated at 25% of the total live load value. This approach recognizes that the maximum design live load on all floors is statistically improbable to occur simultaneously and is based on probabilistic assumptions about structural precision. By implementing this reduced live load part, the code ensures a more economical and realistic design^46^.

**Table 2:** Material properties.

Material	Concrete	Steel
Modulus of elasticity (E) (MPa)	3 × 10^4^	2 × 10^5^
Poisson’s ratio (µ)	0.2	0.3
Compressive strength fc′ (MPa)	30	---
Yield strength (f_y_) (MPa)	---	355
Density (ρ) (kg/m^3^)	2500	7850
Dilation angle (ψ)	31^o^	**---**
Eccentricity(ε)	0.1	**---**
f_b0_/f_c0_	1.16	**---**
K	0.67	**---**
Viscosity parameter	0	---

**Table 3:** Model specifications.

Type of the model	Multi-story steel model
Height of the story	3 m
Number of stories	12 stories
Length of the bay	3 m
Columns sections	HSS 300 × 300 × 12.5
Beams sections	HEB 260
Slab thickness	0.14 m
Damping ratio (ζ)	3%

### FEA setup calibration

**Fig. 2:**
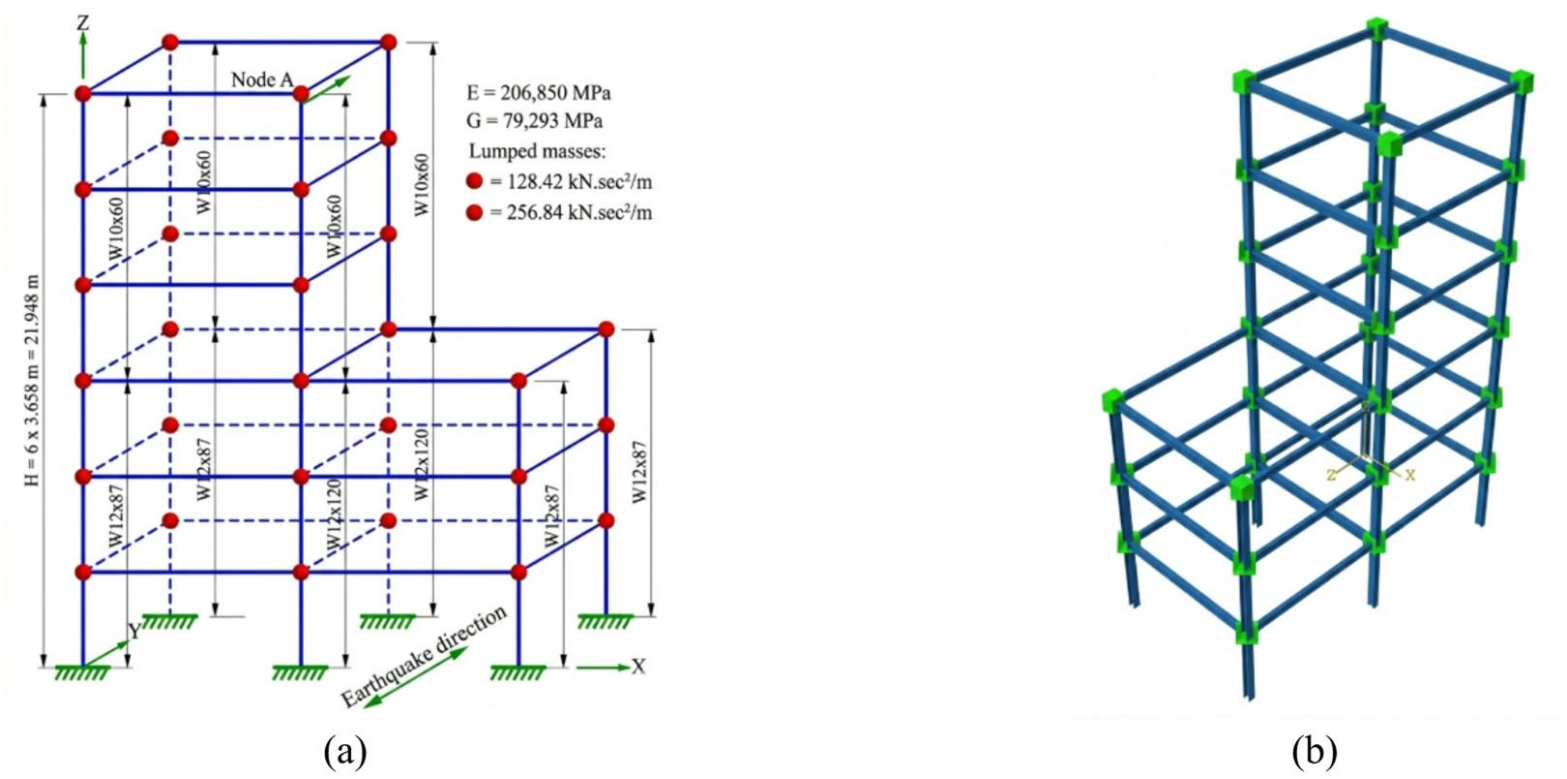
(a) Multi-story steel frame model^47^, (b) ABAQUS Model investigation.

A computational calibration study was performed to ensure the reliability of the solver settings and element formulations. To accomplish this, a benchmark multi-story steel frame previously examined by Nguyen et al. (2021) was reconstructed by SAP 2000. This specific model was chosen because it provides a well-documented baseline for time-history responses under the Loma Prieta earthquake^47^, as shown in Fig. 2.

**Fig. 3:**
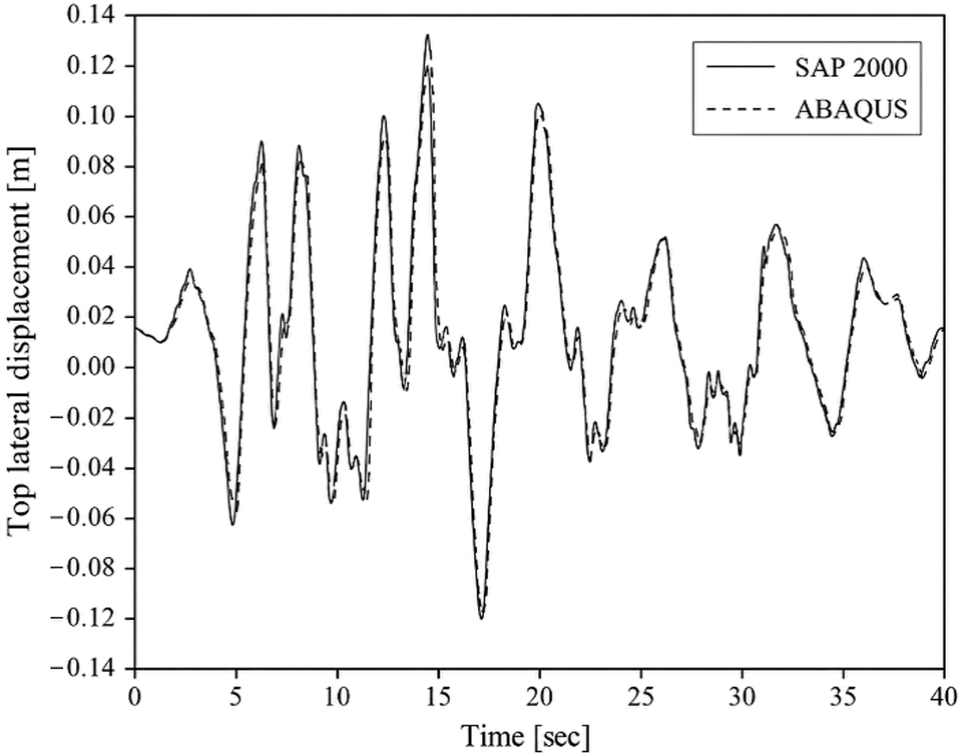
Reference model displacement comparison.

As illustrated in Fig. 3, the comparison between the reference model results and the current finite element simulation shows excellent agreement. The maximum lateral displacement exhibited a marginal variance of less than 5%. Consequently, the established FEA setup can be confidently extended to simulate the complex seismic responses of the 12-story structures presented in the subsequent sections.

### Meshing sensitivity analysis

**Fig. 4:**
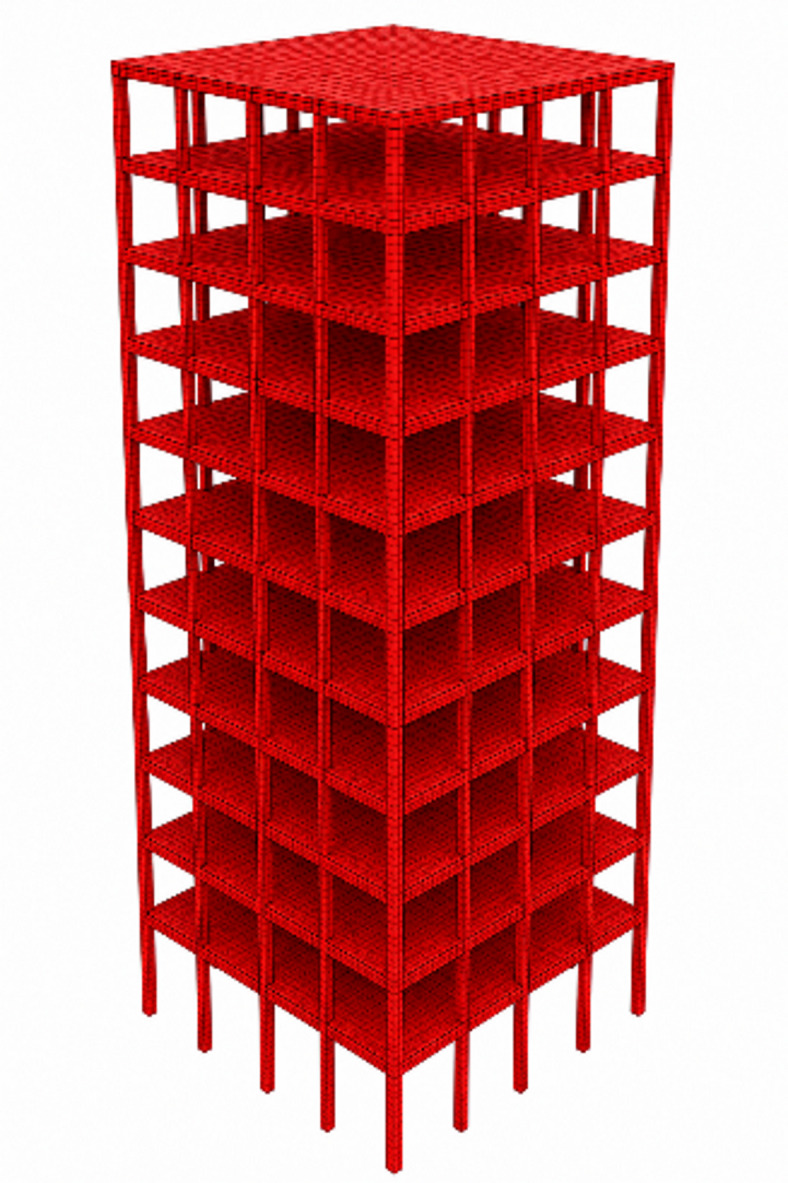
Mesh model.

**Fig. 5:**
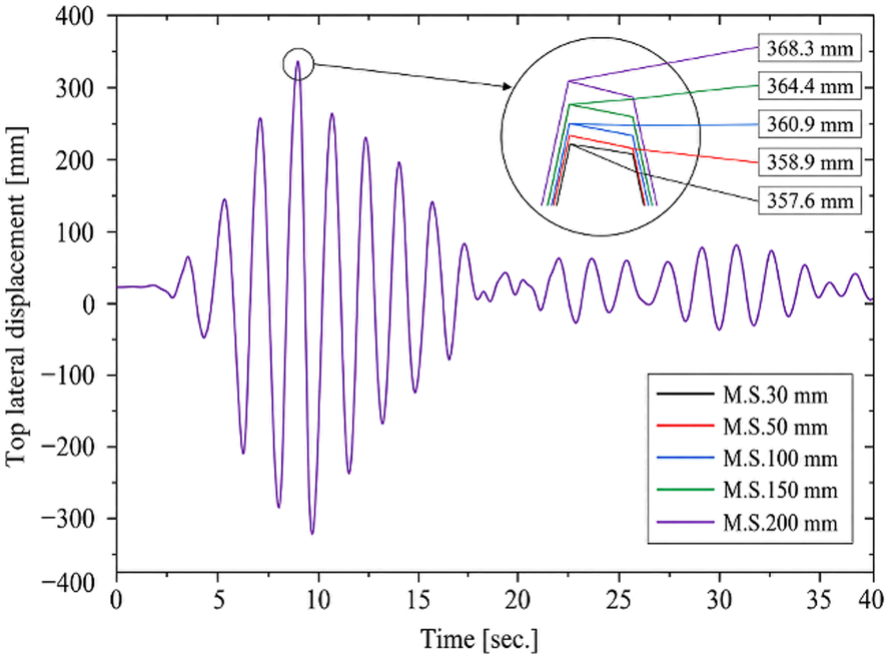
Mesh sensitivity analysis.

The structural model was built using linear 2-node Timoshenko beam elements (B31) for the frame, while the floor diaphragms were represented with 4-node shell elements (S4R)^48^. The intention here was to approximate rigid diaphragm behaviour without overcomplicating the model. To assess whether the results were reliable, a mesh sensitivity study was conducted for the model using five mesh sizes: 30, 50, 100, 150, and 200 mm, as shown in Fig. 4. In the Kobe earthquake record, the course meshes, particularly 150 mm and 200 mm, began to show noticeable differences in top lateral displacement, as shown in Fig. 5. The convergence profile indicated that a 100-mm mesh size provides the optimal balance between computational efficiency and numerical precision. The peak responses were about 1% off from the finer meshes, which means that going smaller wouldn’t be worth the extra computational cost, at least in this case. Based on that, the 100-mm mesh was used for the remaining nonlinear time-history analyses, though one could argue that slightly finer meshes might still be worth testing for more complex geometries.

### Structure modeling

**Fig. 6:**
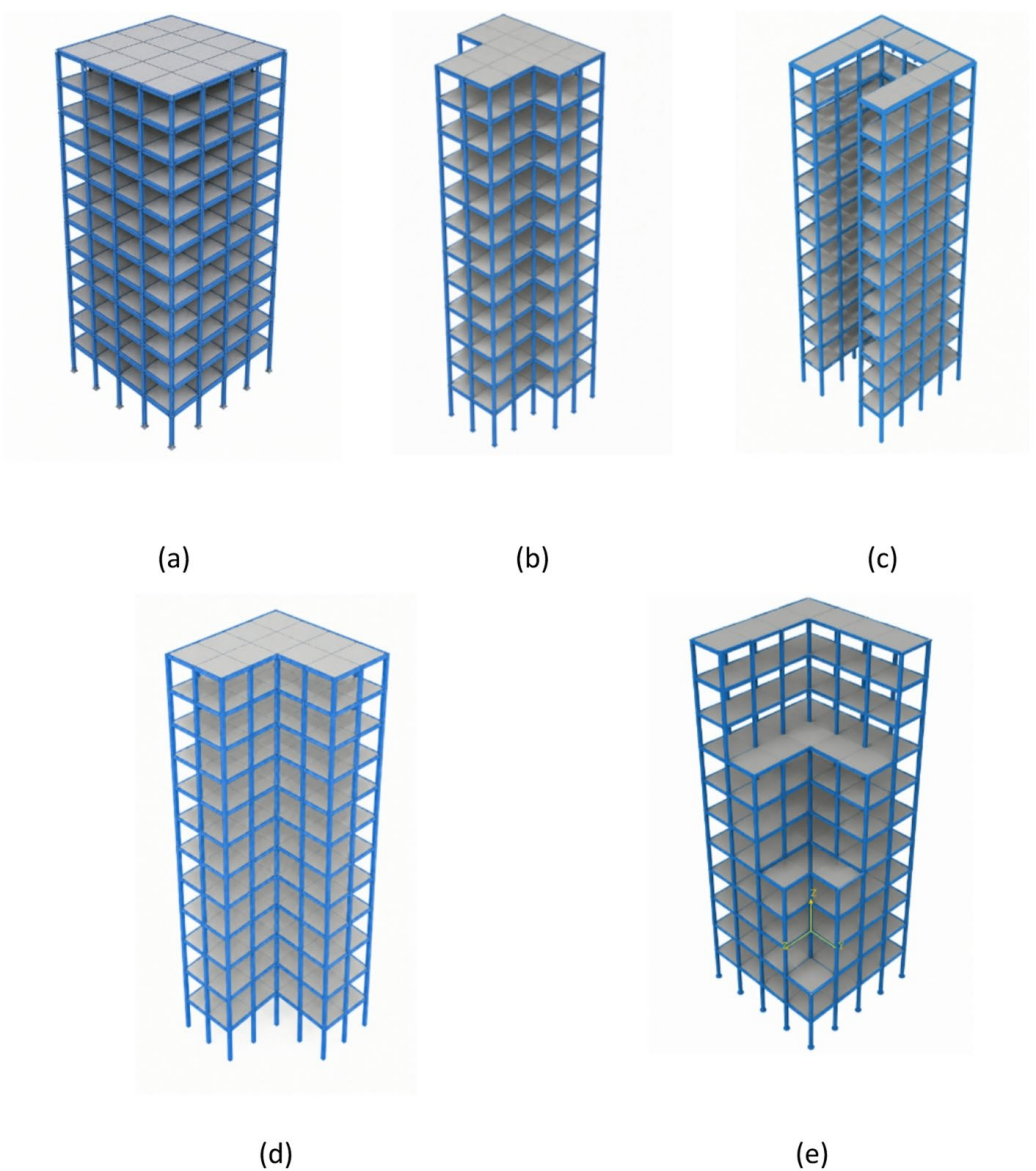
Proposed structural shapes: (a) Regular shape, (b) T-Shape, (c) C-Shape, (d) L-Shape, (e) Stadium shape.

This study evaluated the dynamic response of the proposed models with the efficient distribution of TMDs, including a regular 12-story residential building and other irregular proposed models, such as T, C, L, and stadium shapes, as shown in Fig. 6.

### Modal analysis

Modal analysis was used to estimate the modes of shapes and all related natural circular frequencies of the models to identify critical modes. These critical modes can be used to tune the TMD parameters, as proposed by Sadek et al., to enhance coupling between the building and the TMD and mitigate lateral effects due to seismic excitation^49^. By determining the natural frequencies, the model’s damping is calculated using Rayleigh damping as shown in Table 4, which is a proportional damping coefficient ($\alpha ,\beta )~$based on the model’s mass and stiffness^50^, as illustrated in Eqs. (1–4).

1 $$\left[ c \right]=\alpha \left[ M \right]+\beta \left[ K \right]$$

2 $${\xi _i}=\frac{1}{2}\left( {\frac{\alpha }{{{\omega _i}}}+\beta {\omega _i}} \right)$$

3 $$~\alpha =2\xi \left( {\frac{{~{\omega _m}{\omega _n}}}{{{\omega _m}+{\omega _n}}}} \right)$$

4 $$\beta =\frac{{2\xi }}{{{\omega _m}+{\omega _n}}}$$

**Table 4:** Damping coefficients and natural frequencies.

Modal analysis	Regular 12-story model	Irregular 12-story models			
		T-Shape	C-Shape	L-Shape	Stadium shape
${f_1}$ (Hz)	0.54044	0.54846	0.52999	0.542	0.60567
${f_2}$ (Hz)	0.54048	0.54964	0.60947	0.54862	0.63037
$\alpha$	0.101874	0.10348	0.10686	0.10278	0.11646
$\beta$	0.0088344	0.008697	0.0083811	0.008756	0.007725

### Non-linear time history analysis

To assess how the proposed TMD configurations behave under more realistic seismic conditions, nonlinear response analysis was carried out while accounting for material nonlinearities in both steel and concrete. Steel elements followed an elastic–plastic constitutive model with isotropic hardening^51^, whereas concrete behavior was represented through the Concrete Damaged Plasticity (CDP) formulation, which can reflect stiffness degradation and progressive damage under compression and tension^43^. The interaction between structural nonlinearity and the dynamic properties of the TMD system was also examined, since such nonlinear effects may shift the effective stiffness and natural frequencies of the structure and, in some cases, lead to partial detuning that influences vibration control.

To capture these behaviors, nonlinear time-history analyses were performed using four real earthquake records (Kobe, Chi-Chi, Loma Prieta, and Landers), each representing different frequency characteristics, as outlined in Tables 5 and 6, and all the component orientations are horizontal. These frequency ranges correspond to the standard corrected bandwidths reported in the PEER Ground Motion Database^52^. They were chosen to cover the main energy-carrying part of the spectrum, which, at least in principle, should activate higher structural modes and provide a more realistic assessment of TMD performance under cumulative seismic energy effects.

**Table 5:** Earthquake record information^52^.

Earthquake	Location	Recording station	Component used	Time step Δt (s)	Duration (s)	Frequency range (Hz)
Kobe	Japan, January 16, 1995	KAKOGA`WA(CUE90)	Horizontal	0.01	40	0.1–35
Chi-Chi	Taiwan, September 20, 1999	TCU045	Horizontal	0.01	40	0.02-50
Loma Prieta	USA, October 18, 1989	090 CDMG STATION 47,381	Horizontal	0.01	40	0.1–40
Landers	USA, June 28, 1992	000 SCE STATION 24	Horizontal	0.01	40	0.08-60

**Table 6:** Ground motion characteristics^52^.

Earthquake characteristics	Kobe	Chi-Chi	Loma Prieta	Landers
Max. acceleration (g)	0.344	0.361	0.367	0.78
Max. velocity (cm/sec)	27.6	21.5	44.6	31.5
Max. displacement (cm)	9.694	21.8	19.6	16.5
Acceleration RMS: (g)	0.051	0.02	0.046	0.094
Velocity RMS: (cm/sec)	6.305	4.08	6.796	5.078
Displacement RMS: (cm)	2.294	6.46	3.467	3.64
Characteristic intensity (I_c_)	0.075	0.022	0.064	0.2
Specific energy density (cm^2^/sec)	1625.9	881.1	1843.3	1240.4
Velocity spectrum intensity (cm)	153.5	80.7	169.7	110.1
Effective design acceleration (g)	0.337	0.31	0.359	0.683
A95 parameter (g)	0.227	0.23	0.261	0.471
Predominant period (sec)	0.16	0.06	0.22	0.080
Mean period (sec)	0.542	0.49	0.619	0.165
Max incremental velocity (cm/sec)	43.26	36.34	45.9	29.95
Damage index ((g)^c)	2.786	1.183	2.462	35.44
Number of effective cycles	5.683	2.059	3.546	9.88
IP index	41.78	22.8	20.75	70.79
Sa, avg (g)	0.336	0.187	0.40	0.251

The nonlinear dynamic analyses were performed using the implicit dynamic time-integration procedure in ABAQUS. The earthquake acceleration records were applied with a time increment consistent with the record time step of 0.01 s. The total analysis duration was taken as 40 s for each record. The same time-integration settings, boundary conditions, damping model, and scaled input motion were used for the uncontrolled and controlled cases to ensure consistent comparison, as shown in Table 7.

**Table 7:** Time-integration settings used in nonlinear time-history analysis.

Parameters	Value
Analysis type	Nonlinear dynamic time-history
Time integration	ABAQUS implicit dynamic
Record time step	0.01s
Total duration	40s
Damping model	Rayleigh damping
Boundary condition	Fixed base
Input motion	Scaled horizontal acceleration

### Ground-motion selection and scaling

The selected ground-motion records were obtained from the PEER NGA-West2 database and used in the nonlinear time-history analyses. To ensure a consistent comparison between the uncontrolled and controlled cases, all records were amplitude-scaled to a common intensity measure before being applied to the finite element models. The adopted intensity measure was the 5%-damped spectral acceleration at the fundamental period of the uncontrolled regular 12-story building, S_a_ (T_1_, 5%). This choice was made because the efficiency of TMD systems is strongly related to the dynamic properties of the primary structure.

**Fig. 7:**
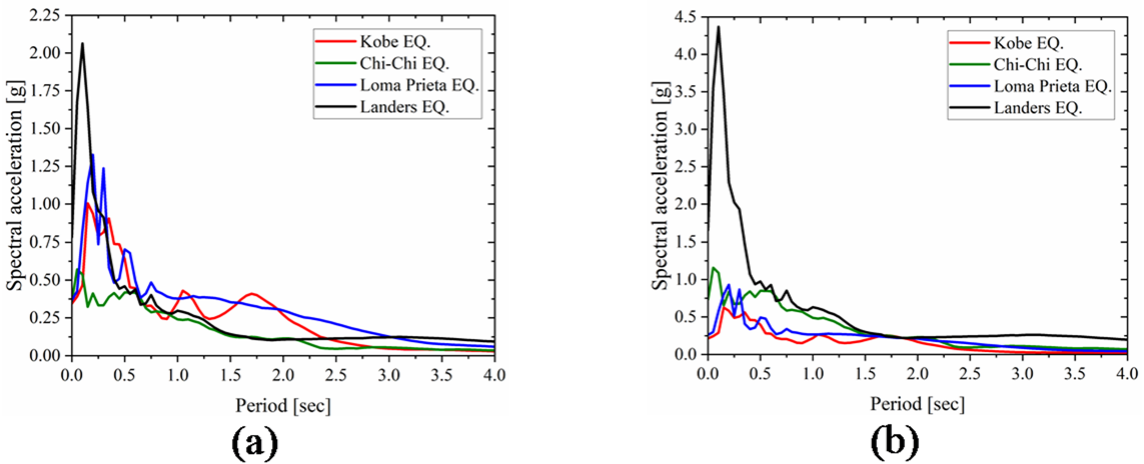
Response spectra of the selected ground motions: (a) Before scaling, and (b) After scaling.

The fundamental period was obtained from modal analysis as T₁ = 1.85 s. The target value was selected as S_a_ (T_1_, 5%) = 0.2205 g, and the scale factors were calculated using Eqs. (5–7). The original spectral acceleration, target spectral acceleration, scale factor, and scaled intensity value for each record are reported in Table 8. The acceleration response spectra before and after scaling are shown in Fig. 7.

**Fig. 8:**
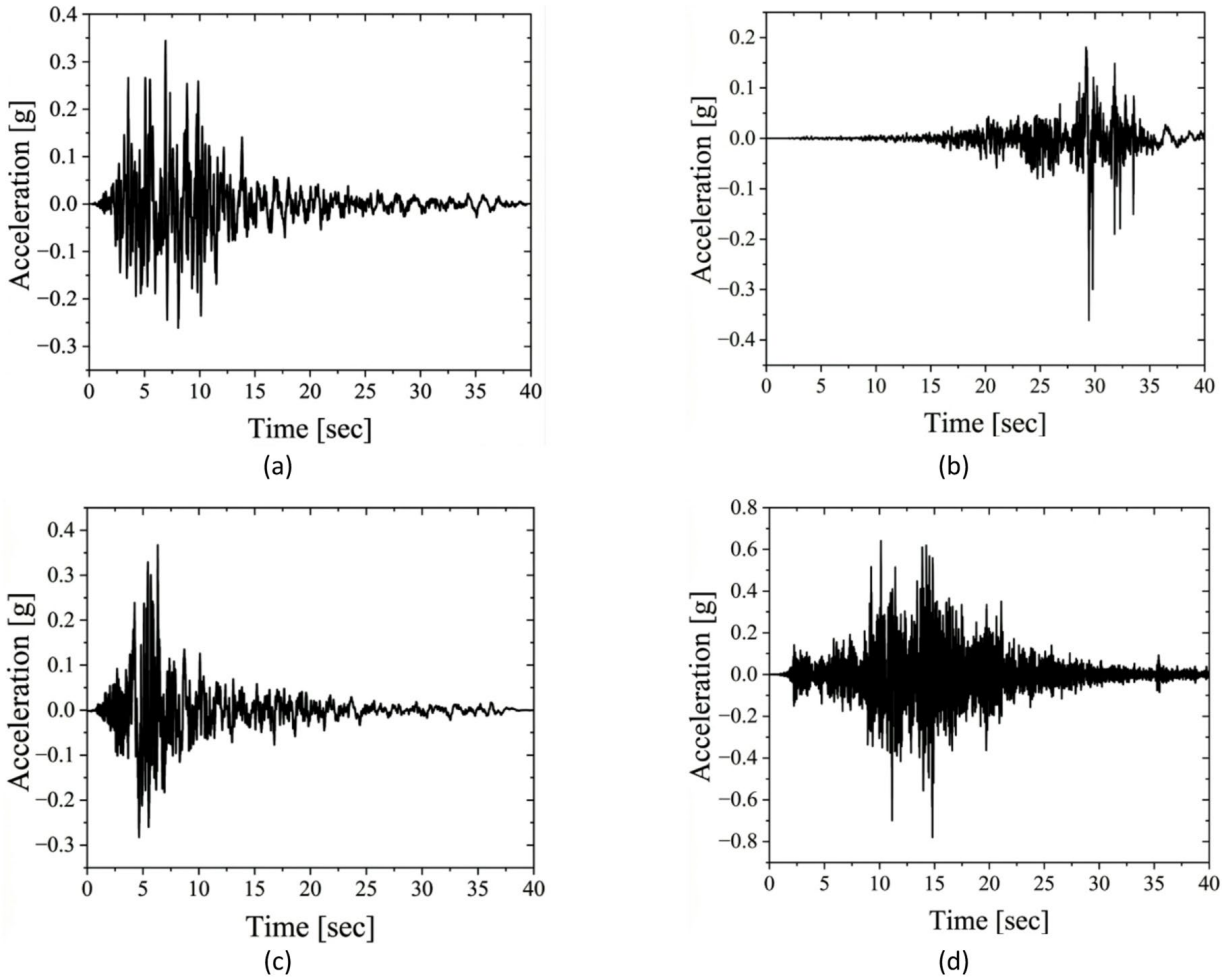
Acceleration time histories of the selected records: (a) Kobe, (b) Chi-Chi, (c) Loma Prieta, and (d) Landers.

The scaled spectra indicate that the records were adjusted to the same target S_a_ (T_1_, 5%) at the fundamental period while retaining their different frequency-content characteristics. No spectrum matching was applied to preserve the original phase characteristics, nonstationary frequency content, and time-domain features of the recorded accelerograms. The scaled acceleration time histories used in the analyses are shown in Fig. 8. For each structural configuration, the same horizontal component orientation, scale factor, and input motion were used for the uncontrolled and controlled cases. Therefore, the reported response reductions represent direct comparisons between TMD configurations under consistently scaled records. Although all records were scaled to a common Sa (T₁, 5%) intensity level, TMD performance remains sensitive to frequency content, duration, pulse characteristics, and excitation direction. Therefore, the reported reductions are interpreted as comparisons among the investigated TMD configurations under the selected scaled records.

5 $$S{F_i}~=~\frac{{{S_{a,target}}\left( {{T_1},5\% } \right)}}{{{S_{a,i}}\left( {{T_1},5\% } \right)}}$$

6 $$~~~~~{S_{a,target}}\left( {{T_1},5\% } \right)~=~median\left[ {{S_{a,i}}\left( {{T_1},5\% } \right)} \right]$$

7 $${S_{scaled,i}}\left( t \right)~=~S{F_i}~{S_i}\left( t \right)$$

**Table 8:** Scaling parameters of the earthquake records.

Earthquake	S_a_ (T_1_, 5%) (g)	Target S_a_ (T_1_, 5%) (g)	Scale factor (SF)	Scaled S_a_ (T_1_, 5%) (g)
Kobe	0.3551	0.2205	0.621	0.2205
Chi-Chi	0.10874	0.2205	2.028	0.2205
Loma Prieta	0.31415	0.2205	0.702	0.2205
Landers	0.10416	0.2205	2.117	0.2205

## FEA setup of the TMD

A TMD is a passive control system containing a mass that represents 2 to 5% of the building’s total mass, which is linked to the structure via a spring and a viscous damper. Their parameters include the viscous damping coefficient and stiffness, which can be calculated using many tuning techniques to optimize the coupling between the TMD and the main system to dissipate the lateral response caused by seismic effects. In this research, the parameters of TMD were determined in Table 9 using the Sadek et al. method^49^, which is more accurate because it accounts for the damping ratio in the calculation, as illustrated in Eqs. (8–11).

8 $$\mu =\frac{{{m_{TMD}}}}{{{m_S}}}$$

9 $${f_{opt}}.~=\frac{1}{{1+\mu }}\left[ {1 - \xi \sqrt {\frac{\mu }{{1+\mu }}} ~} \right]~=\frac{{{\omega _{TMD,opt.}}}}{{{\omega _S}}}$$

10 $${\xi _{TMD,opt.}}=\left[ {~\frac{\xi }{{1+\mu }}+\sqrt {\frac{\mu }{{1+\mu }}} ~} \right]=\frac{{{C_{TMD,opt.}}}}{{2{m_{TMD}}{\omega _{TMD,opt.}}}}$$

11 $${k_{TMD,opt.}}={m_{TMD}}{\left( {{\omega _{TMD,opt.}}} \right)^2}$$

**Table 9:** TMD design parameters.

Parameters	Regular 12-story model					Irregular 12-story models			
						MTMDs			
	STMD				MTMDs	T-Shape	C-Shape	L-Shape	Stadium shape
No. of TMDs	1	1	1	1	4	4	4	5	5
${{\mathrm{\boldsymbol{\upmu}}}_{{\mathrm{total}}}}$	2%	3%	4%	5%	5%	5%	5%	5%	5%
${{\mathrm{m}}_{\mathrm{s}}}$ (ton)	2290.38	2290.38	2290.38	2290.38	2290.38	1760	1532.6	1773.6	1840
${{\mathrm{\boldsymbol{\upomega}}}_{\mathrm{S}}}$ (rad/sec.)	3.396	3.396	3.396	3.396	3.396	3.446	3.33	3.404	3.805
${{\mathrm{\boldsymbol{\upomega}}}_{{\mathrm{TMD}}}}$(rad/sec.)	3.314	3.281	3.246	3.213	3.213	3.26	3.151	3.22	3.599
${{\mathrm{m}}_{{\mathrm{TMD}}}}$(ton)	45.808	68.711	91.615	114.52	28.63	22	19.158	17.74	18.4
${{\mathrm{k}}_{{\mathrm{TMD}}}}$(N/mm)	503.45	739.223	965.303	1182.22	295.558	233.81	190.211	183.894	238.332
${{\mathrm{C}}_{{\mathrm{TMD}}}}$(N/mm)	51.451	90.06	133.822	181.62	45.41	35.4	29.796	28.212	32.713
${{\mathrm{\boldsymbol{\upxi}}}_{{\mathrm{TMD}}}}$	0.169	0.199	0.225	0.247	0.247	0.247	0.247	0.247	0.247

## Configuration distribution of TMD in the regular and irregular models

This section describes the selected TMD configurations investigated to compare the influence of mass ratio, vertical distribution, horizontal distribution, slight detuning, and perimeter-based placement on the seismic response of the studied buildings. Initially, an STMD was evaluated on the top floor of a 12-story regular steel building using varying mass ratios (2%, 3%, 4%, and 5%) across a diverse suite of earthquake records to establish a baseline for seismic response mitigation. A total mass ratio of 5% was then subdivided into multiple units to examine how different spatial arrangements influence the response of the selected models. Vertically, the mass was equally partitioned among four MTMD units distributed across the lower, middle, and upper thirds, as well as a strategic ‘selective floor’ arrangement (stories 1, 4, 8, and 12). Furthermore, a horizontal distribution was investigated by positioning the units at the building corners. This strategy was extended to irregular structural geometries, in which the MTMDs were deployed along the outer perimeter relative to the center of rigidity.

### Dynamic eccentricity and MTMD location coordinates

**Fig. 9:**
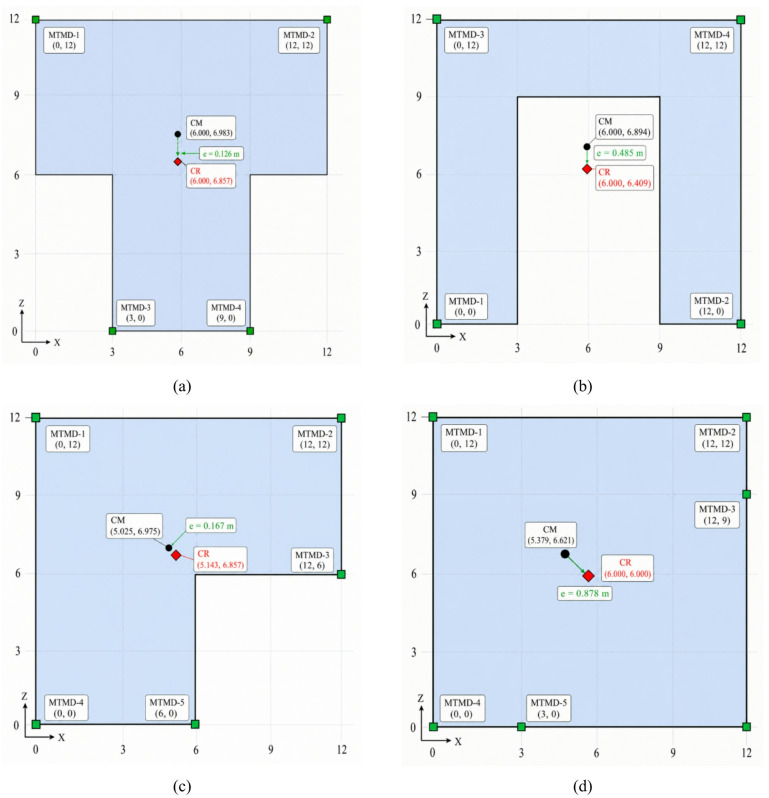
MTMD location coordinates and dynamic eccentricity for the irregular plan configurations: (a) T-shape, (b) C-shape, (c) L-shape, and (d) Stadium shape.

For each irregular plan configuration, the center of mass (CM), center of rigidity (CR), and structural eccentricity were quantified in the same X-Z coordinate system used for the finite element models. The CM was obtained from the mass-weighted coordinates of the floor masses, whereas the CR was evaluated from the stiffness-weighted coordinates of the vertical lateral-resisting lines. The offset between CM and CR defines the eccentricity vector responsible for coupled translational-torsional response under seismic excitation, which was determined as illustrated in Eqs. (12–18). Therefore, the MTMD coordinates were selected at perimeter positions that provide large torsional lever arms relative to the CR that could be calculated as shown in Eq. (19). Tables 10 and 11 show the dynamic eccentricity and MTMD location coordinates relative to CR, as illustrated in Fig. 9.

12 $${x_{CM}}=\frac{{\sum {m_i}{x_i}}}{{\sum {m_i}}}$$

13 $${z_{CM}}=\frac{{\sum {m_i}{z_i}}}{{\sum {m_i}}}$$

14 $${x_{CR}}=\frac{{\sum {k_i}{x_i}}}{{\sum {k_i}}}$$

15 $${z_{CR}}=\frac{{\sum {k_i}{z_i}}}{{\sum {k_i}}}$$

16 $${e_x}={c_{CM}} - {x_{CR}}$$

17 $${e_z}={z_{CM}} - {z_{CR}}$$

18 $$e=\sqrt {\left( {e_{x}^{2}+e_{z}^{2}} \right)}$$

19 $${r_{TMD}}=\sqrt {{{\left( {{x_{TMD}} - {x_{CR}}} \right)}^2}+{{\left( {{z_{TMD}} - {z_{CR}}} \right)}^2}}$$

**Table 10:** Dynamic eccentricity for the irregular plan configurations.

Plan	CM (x, z) m	CR (x, z) m	e_x_ (m)	e_z_ (m)	e (m)
T-shape	(6.000, 6.983)	(6.000, 6.857)	0.000	0.126	0.126
C-shape	(6.000, 6.894)	(6.000, 6.409)	0.000	0.485	0.485
L-shape	(5.025, 6.975)	(5.143, 6.857)	-0.118	0.118	0.167
Stadium shape	(5.379, 6.621)	(6.000, 6.000)	-0.621	0.621	0.878

**Table 11:** MTMD location coordinates and torsional lever arms relative to the CR for each plan configuration.

Plan	MTMD	Coordinate (x, z) m	*r*_TMD_ from CR (m)
T-shape	TMD 1	(6.00, 6.857)	0.000
	TMD 2	(0.0, 12.0)	7.902
	TMD 3	(12.0, 12.0)	7.902
	TMD 4	(3.0, 0.0)	7.485
C-shape	TMD 1	(0.0, 0.0)	8.779
	TMD 2	(12.0, 0.0)	8.779
	TMD 3	(0.0, 12.0)	8.201
	TMD 4	(12.0, 12.0)	8.201
L-shape	TMD 1	(0.0, 12.0)	7.273
	TMD 2	(12.0, 12.0)	8.571
	TMD 3	(12.0, 6.0)	6.911
	TMD 4	(0.0, 0.0)	8.571
	TMD 5	(6.0, 0.0)	6.911
Stadium shape	TMD 1	(9.0, 0.0)	7.485
	TMD 2	(0.0, 12.0)	8.485
	TMD 3	(12.0, 12.0)	8.485
	TMD 4	(12.0, 9.0)	6.708
	TMD 5	(0.0, 0.0)	8.485

**Fig. 10:**
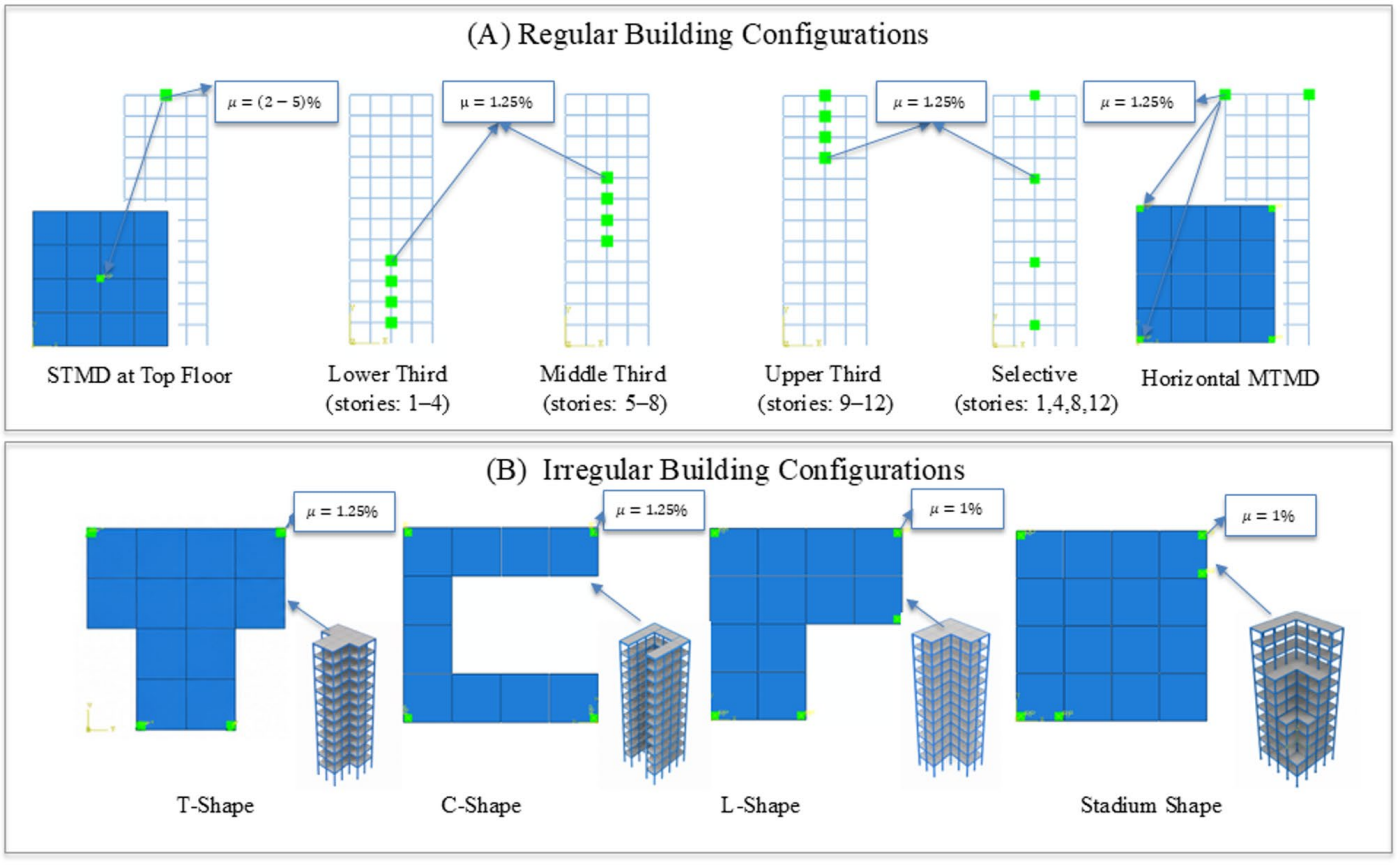
Schematic illustration of the investigated TMD configurations: (A) Regular MSB including STMD at top floor, vertical MTMD distributions (Lower, Middle, Upper thirds, and Selective stories), and horizontal corner placement; (B) Irregular MSBs showing perimeter-based MTMD distribution for T, C, L, and Stadium shapes.

The MTMD locations were selected based on a coordinate-based interpretation of the CM–CR eccentricity for each irregular plan. In all models, the dampers were placed at boundary or corner regions to maximize their torsional lever arms relative to the CR and to improve their contribution to controlling coupled translational–torsional response. For the C- and T-shaped models, the MTMD layout follows the main symmetry characteristics of the plan, while addressing the dominant Z-direction eccentricity. In the L-shaped model, five boundary dampers were used to cover both the upper flange and the lower re-entrant leg, with the largest lever arms occurring at diagonally opposite perimeter points. For the stadium-shaped model, the MTMDs were distributed around the CM–CR eccentricity vector to provide balanced control. Therefore, the selected MTMD positions are not arbitrary but are directly linked to the calculated eccentricity and the need to enhance torsional response mitigation in irregular structures. The investigated configurations are summarized schematically in Fig. 10 and were then evaluated through nonlinear time-history analysis.

## Numerical analysis of TMD in the regular model

### STMD on the top floor

**Fig. 11:**
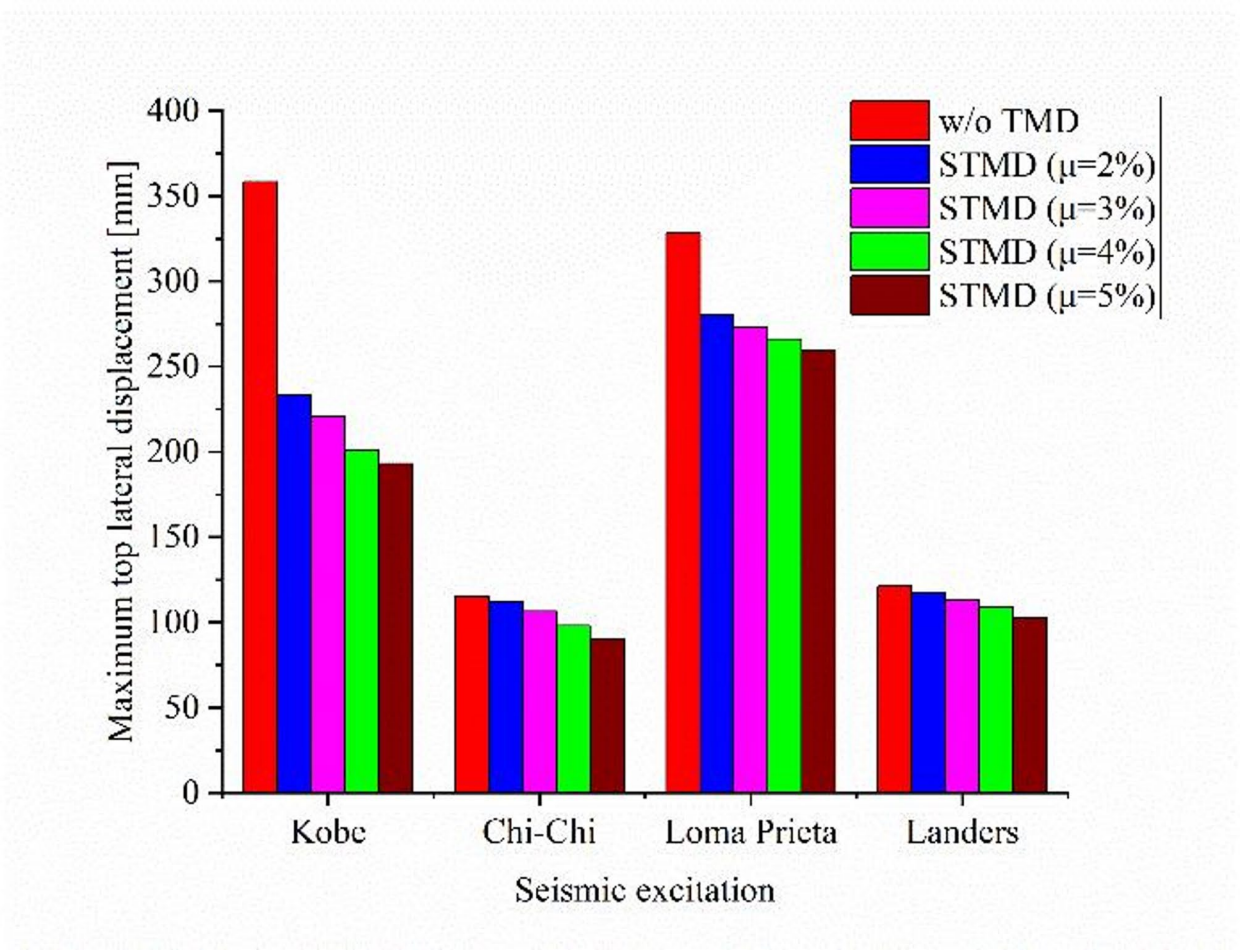
Maximum top lateral displacement of the building with and without STMD on the top floor with mass ratios of 2%, 3%, 4%, and 5% under the selected earthquake excitations.

A numerical simulation of a TMD installed on the top floor of a regular multi-story steel building was conducted in ABAQUS for four real-time earthquake scenarios with varying TMD-to-building mass ratios. The simulation assessed the building’s seismic response, with particular focus on the maximum top lateral displacement, as illustrated in Fig. 11.

**Fig. 12:**
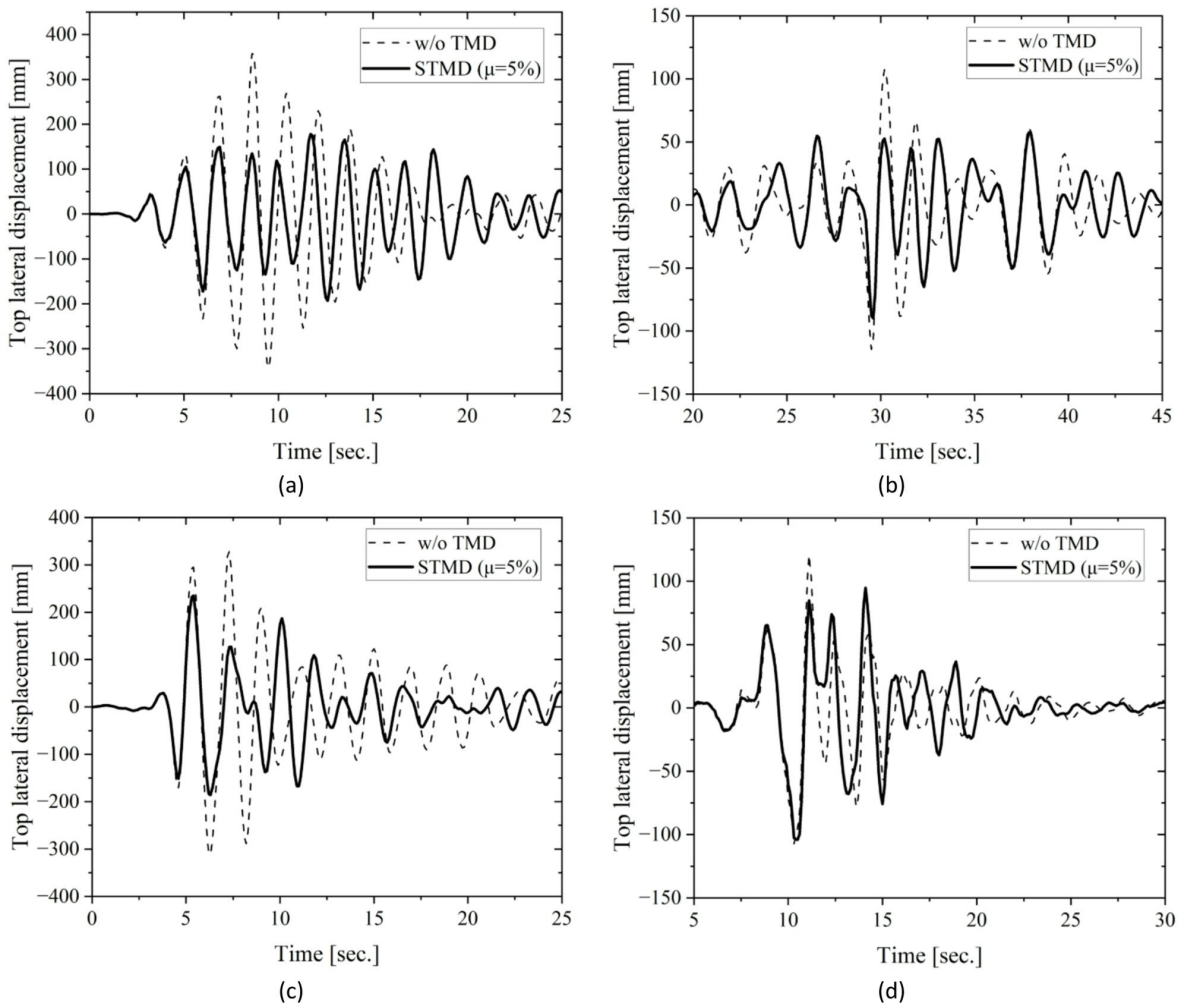
Top lateral displacement time histories of the regular building with and without STMD under (a) Kobe, (b) Chi-Chi, (c) Loma Prieta, and (d) Landers earthquake excitation.

The modal analysis of the regular 12-story steel building showed that its dominant natural frequencies were within the frequency ranges of the Kobe and Loma Prieta records. Accordingly, these records produced a higher maximum top lateral displacement than the other selected records. The corresponding time-history responses are shown in Fig. 12, where the displacement histories with and without STMD are compared under the four selected earthquake records. Installing an STMD on the top floor reduced the lateral response under the considered seismic excitations. Increasing the TMD-to-building mass ratio also reduced the maximum top lateral displacement; however, concentrating a larger auxiliary mass at a single floor may require additional checks related to load transfer and installation, which were not quantified in the present study. The effectiveness of the STMD was also influenced by the frequency content of the input records. At a 5% mass ratio, the maximum top lateral displacement was reduced by 46.11%, 21.74%, 21.0%, and 14.88% under the Kobe, Chi-Chi, Loma Prieta, and Landers records, respectively, as summarized in Fig. 11. These results indicate that the response-reduction efficiency of the STMD was record-dependent, even after scaling the ground motions to a common Sa (T_1_, 5%) intensity level.

### MTMDs distributed vertically

**Fig. 13:**
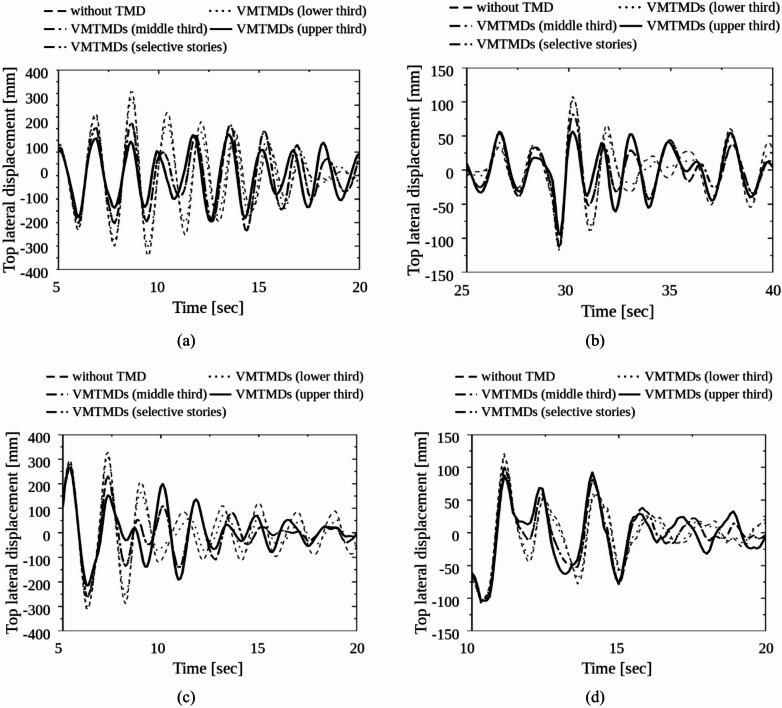
Top lateral displacement time histories of the regular building with and without vertically distributed MTMDs under (a) Kobe, (b) Chi-Chi, (c) Loma Prieta, and (d) Landers earthquake excitation.

The vertical multiple tuned mass damper (VMTMD) was divided into four masses, which were placed in the lower third, middle third, and upper third of the model, as well as in selected stories throughout the entire structure, to evaluate their effectiveness in mitigating lateral displacement during seismic events, as shown in Fig. 13.

**Fig. 14:**
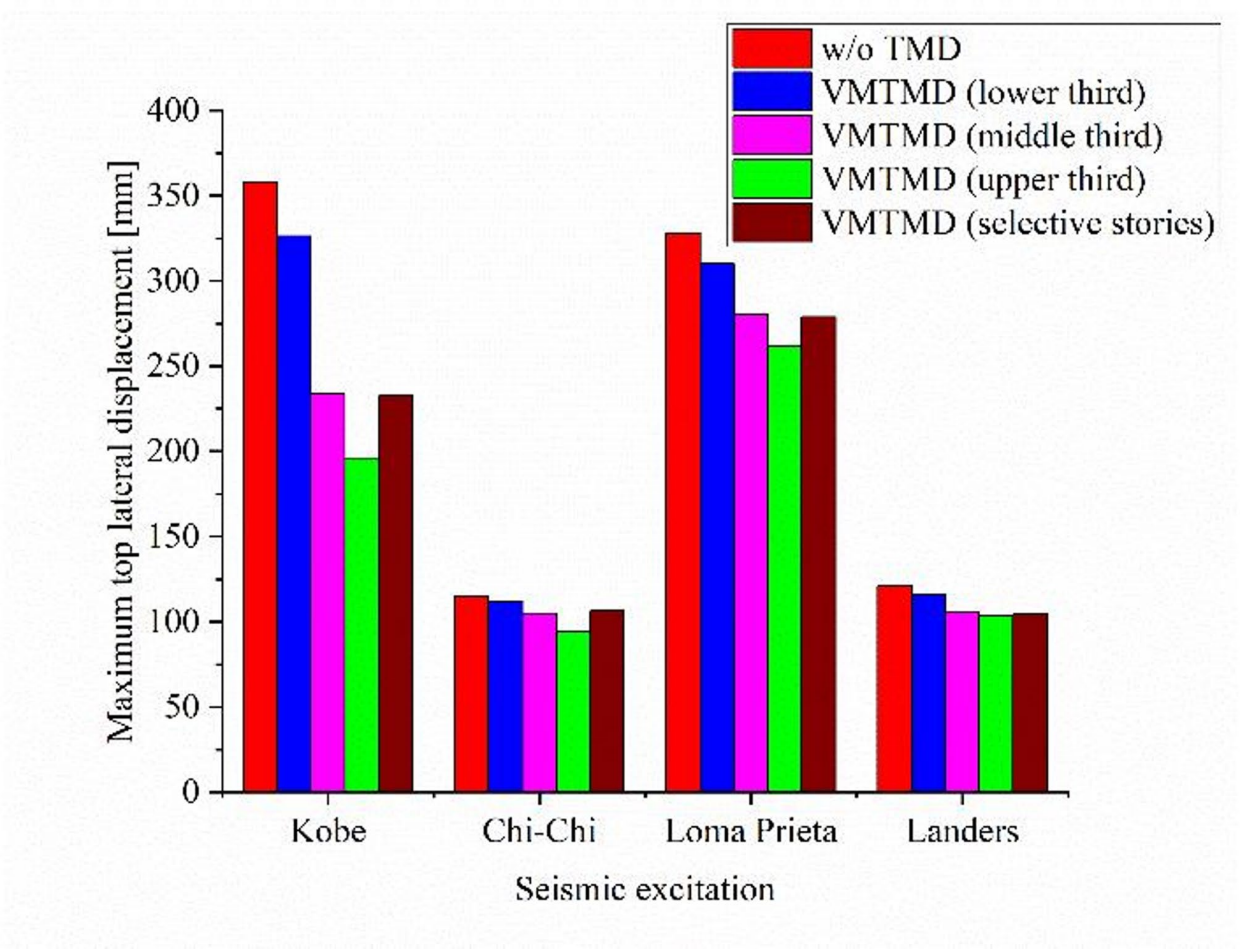
Maximum top lateral displacement of the regular building for the investigated vertical MTMD layouts under the selected earthquake records.

Among the investigated vertical MTMD layouts, the upper-third distribution produced the largest reduction in maximum top lateral displacement, reaching 45.3%, 18.26%, 20.3%, and 14.5% under the Kobe, Chi-Chi, Loma Prieta, and Landers records, respectively, as shown in Fig. 14. The selective-story arrangement showed a different response pattern along the height, but it was not consistently superior in terms of peak top displacement. Therefore, the vertical-distribution results are reported as configuration-specific comparisons under the selected records, rather than as a general recommendation for all building heights or earthquake inputs.

### Horizontally distributed MTMDs

**Fig. 15:**
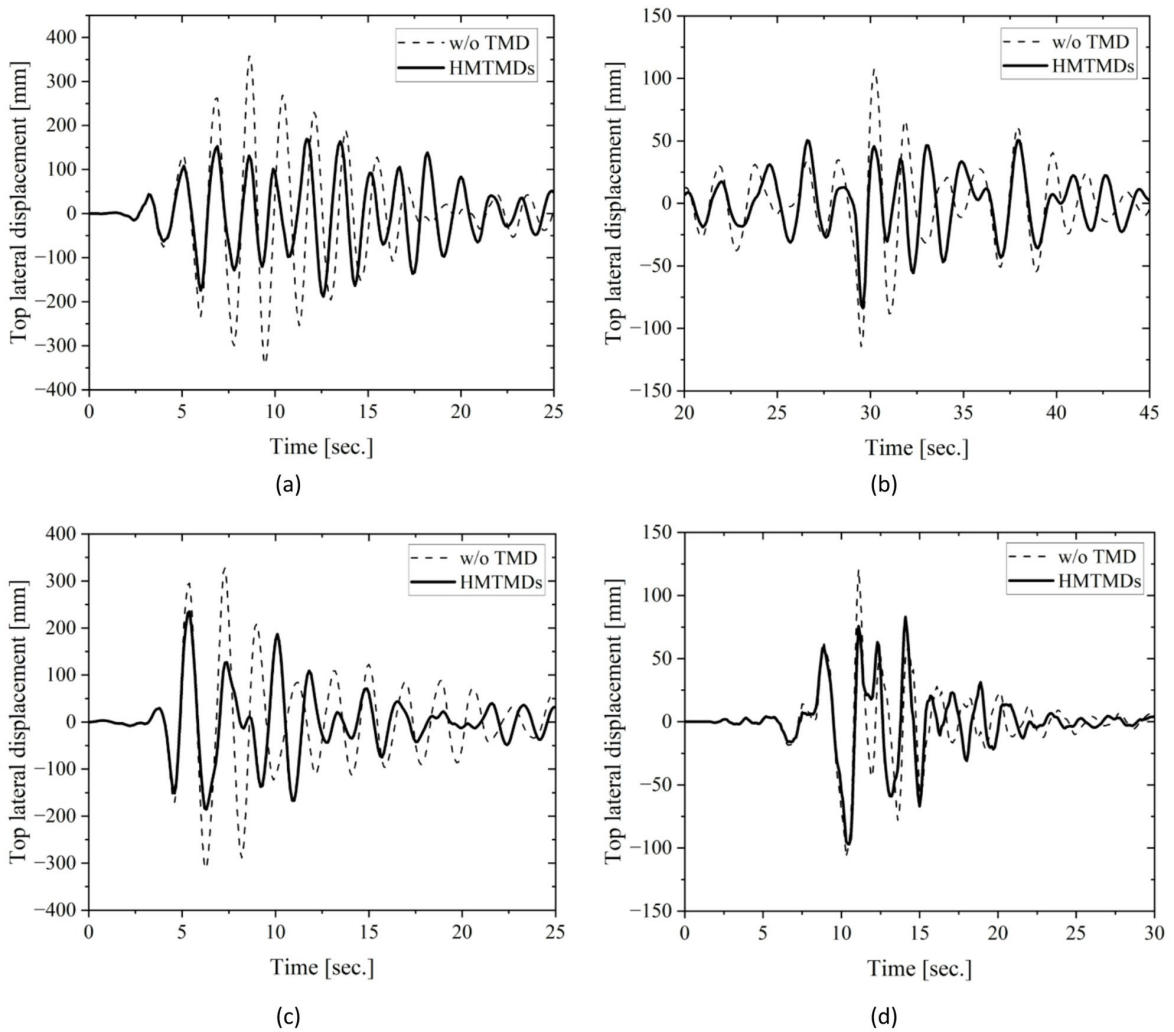
Top lateral displacement time histories of the regular building with and without the horizontal four-corner MTMD layout under (a) Kobe, (b) Chi-Chi, (c) Loma Prieta, and (d) Landers earthquake excitation.

The total auxiliary mass corresponding to a 5% mass ratio was divided into four horizontal multi-tuned mass dampers (HMTMD) units and placed at the four corners of the top floor of the regular steel building. This layout was examined to evaluate whether distributing the same total auxiliary mass at the roof corners could reduce the lateral response compared with concentrating it in a single roof-level STMD. The resulting top lateral displacement responses are shown in Fig. 15.

**Fig. 16:**
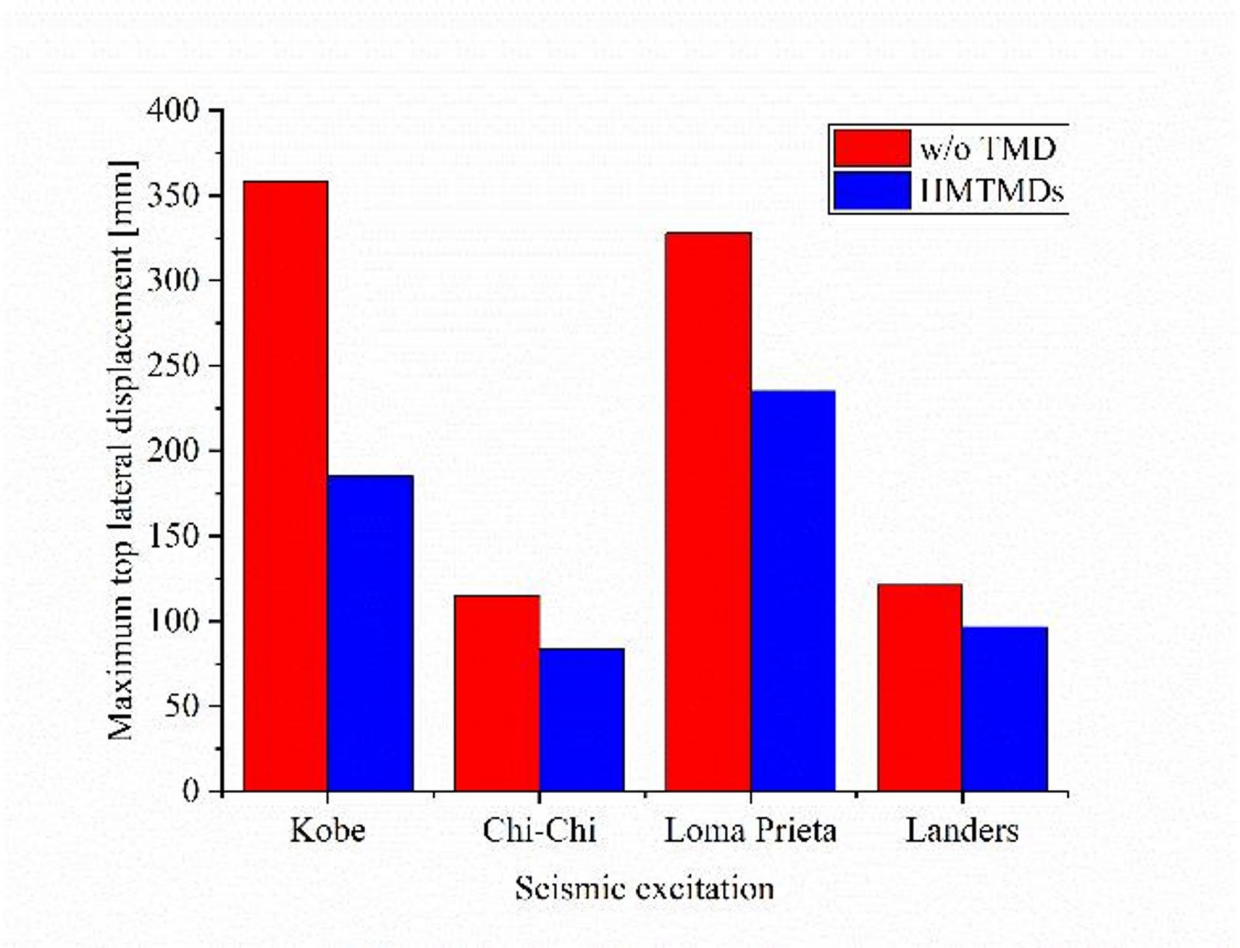
Maximum top lateral displacement of the regular building with and without the horizontal four-corner MTMD layout under the selected earthquake records.

The four-corner MTMD configuration reduced the maximum top lateral displacement by 48.34%, 27.4%, 28.35%, and 20.25% under the Kobe, Chi-Chi, Loma Prieta, and Landers records, respectively, as shown in Fig. 16. These results indicate that, for the selected regular building and scaled records, distributing the same total auxiliary mass at the roof corners produced lower peak displacement than the uncontrolled case.

### Additional engineering demand parameters for the regular-building TMD layouts

Engineering demand parameters (EDPs) were extracted for the regular 12-story building under the four scaled earthquake records. These parameters include maximum top-floor displacement, inter-story drift displacement, floor acceleration, base shear, overturning moment, TMD stroke, and damper force. The purpose of this comparison is to report these response quantities for STMD, vertically distributed MTMDs at different height ranges, the selective-story MTMD arrangement, and the four-corner horizontal MTMD layout. The peak responses under Kobe, Chi-Chi, Loma Prieta, and Landers excitations are summarized in Tables 12, 13, 14 and 15, respectively. The results are used to broaden the comparison of responses within the selected models and records.

**Table 12:** EDPs of the 12-story regular model with various TMD distributions under Kobe excitation.

Peak response	Without TMD	STMD ($\mu =5\%$)	VMTMD (lower third)	VMTMD (middle third)	VMTMD (upper third)	VMTMD (selective stories)	HMTMD ($\mu =5\%$)
Maximum top-floor displacement, ${U_{floor}}$(mm)	357.494	196	326.342	233.469	195.469	233.426	185
Maximum inter-story relative displacement (mm)	42.4	28.9	36.7	32.6	29.3	32.9	26.4
Maximum top-floor acceleration, ${A_{floor}}$(mm/s_2_)	5926	4591.72	5593.08	4586.56	4467.14	4581.32	4376.28
Base shear (kN)	195.405	108.767	185.384	134.973	105.226	130	107.8
Over-turning moment (kN · m)	418.825	232.908	394.185	286.013	225.475	276	230.8
Damper stroke (mm)	-	708.187	640.398	823.279	758.823	835.67	692.5
Damper force (kN)	-	868.913	201.01	249.86	231.113	311	216

**Table 13:** EDPs of the 12-story regular model with various TMD distributions under Chi-Chi excitation.

Peak response	W/o	STMD ($\mu =5\%$)	VMTMD (lower third)	VMTMD (middle third)	VMTMD (upper third)	VMTMD (selective stories)	HMTMD ($\mu =5\%$)
Maximum top-floor displacement, ${U_{floor}}$(mm)	114.505	90	110.71	101.34	89.25	102.14	83.5
Maximum inter-story relative displacement (mm)	14.92	10.45	12.84	11.56	10.62	11.69	9.68
Maximum top-floor acceleration, ${A_{floor}}$(mm/s_2_)	2720	2630.48	2702.19	2675.46	2632.41	2672.61	2507.22
Base shear (kN)	74.348	59.458	71.55	66.38	60.68	65.94	57.34
Over-turning moment (kN · m)	158.402	125.822	151.98	138.68	123.87	139.46	123.78
Damper stroke (mm)	-	241.263	236.48	254.64	244.65	256.14	233.45
Damper force (kN)	-	292.448	68.85	76.56	74.46	79.84	71.24

**Table 14:** EDPs of the 12-story regular model with various TMD distributions under Loma Prieta excitation.

Peak response	W/o	STMD ($\mu =5\%$)	VMTMD (lower third)	VMTMD (middle third)	VMTMD (upper third)	VMTMD (selective stories)	HMTMD ($\mu =5\%$)
Maximum top-floor displacement, ${U_{floor}}$(mm)	328	259	306.84	288.34	261.5	287.24	235
Maximum inter-story relative displacement (mm)	37.2	30.12	35.48	33.15	30.46	33.57	27.8
Maximum top-floor acceleration, ${A_{floor}}$(mm/s²)	5383.37	4996.5	5268.75	5123.67	5014.35	5125.36	4725.73
Base shear (kN)	182.799	159.338	178.65	168.34	160.13	169.11	157.758
Over-turning moment (kN · m)	389.477	340.132	374.55	356.87	341.65	357.24	339.02
Damper stroke (mm)	-	807.856	744.3	832.656	814.5	836.2	752.46
Damper force (kN)	-	1166.5	272.36	296.35	291.6	306.4	288.372

**Table 15:** EDPs of the 12-story regular model with various TMD distributions under Landers excitation.

Peak response	W/o	STMD ($\mu =5\%$)	VMTMD (lower third)	VMTMD (middle third)	VMTMD (upper third)	VMTMD (selective stories)	HMTMD ($\mu =5\%$)
Maximum top-floor displacement, ${U_{floor}}$(mm)	121	103	118.3	111.4	103.5	103.8	96.5
Maximum inter-story relative displacement (mm)	13.4	12.15	13.1	12.75	12.23	12.84	11.52
Maximum top-floor acceleration, ${A_{floor}}$(mm/s²)	2831	2746.3	2806.4	2776.5	2752.6	2781.3	2639.4
Base shear (kN)	72.8	69.85	72.14	71.46	70.1	71.68	68.23
Over-turning moment (kN · m)	153.163	147.61	151.79	149.88	147.83	150.24	146.74
Damper stroke (mm)	-	247.2	241.65	253.6	250.3	257.46	243.6
Damper force (kN)	-	360.33	82.35	87.46	86.42	89.85	85.34

**Fig. 17:**
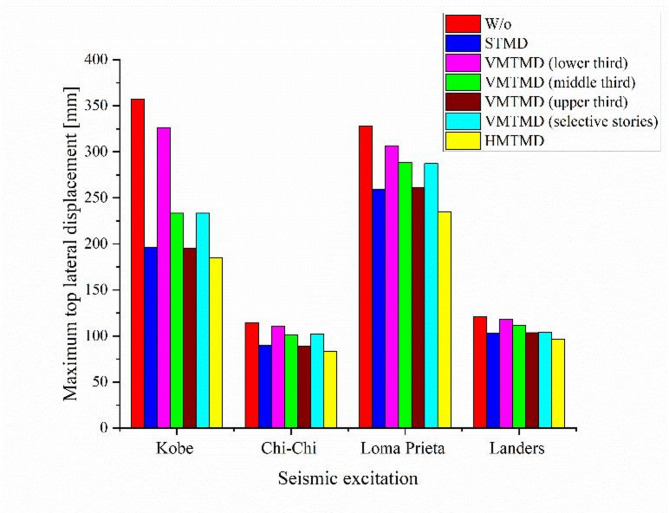
Maximum top-floor displacement for the investigated TMD layouts under the four scaled earthquake records.

**Fig. 18:**
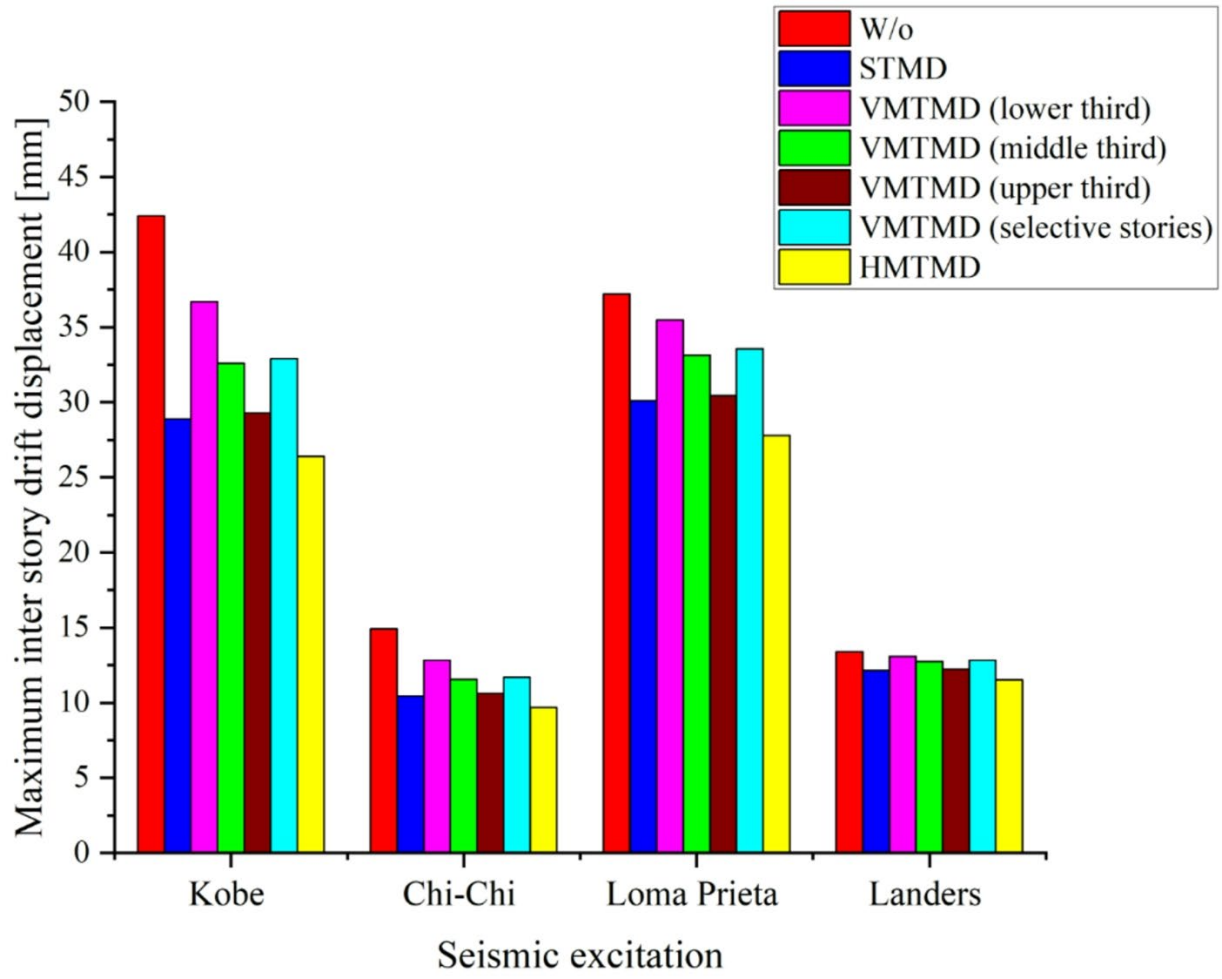
Maximum inter-story drift displacement for the investigated TMD layouts under the four scaled earthquake records.

**Fig. 19:**
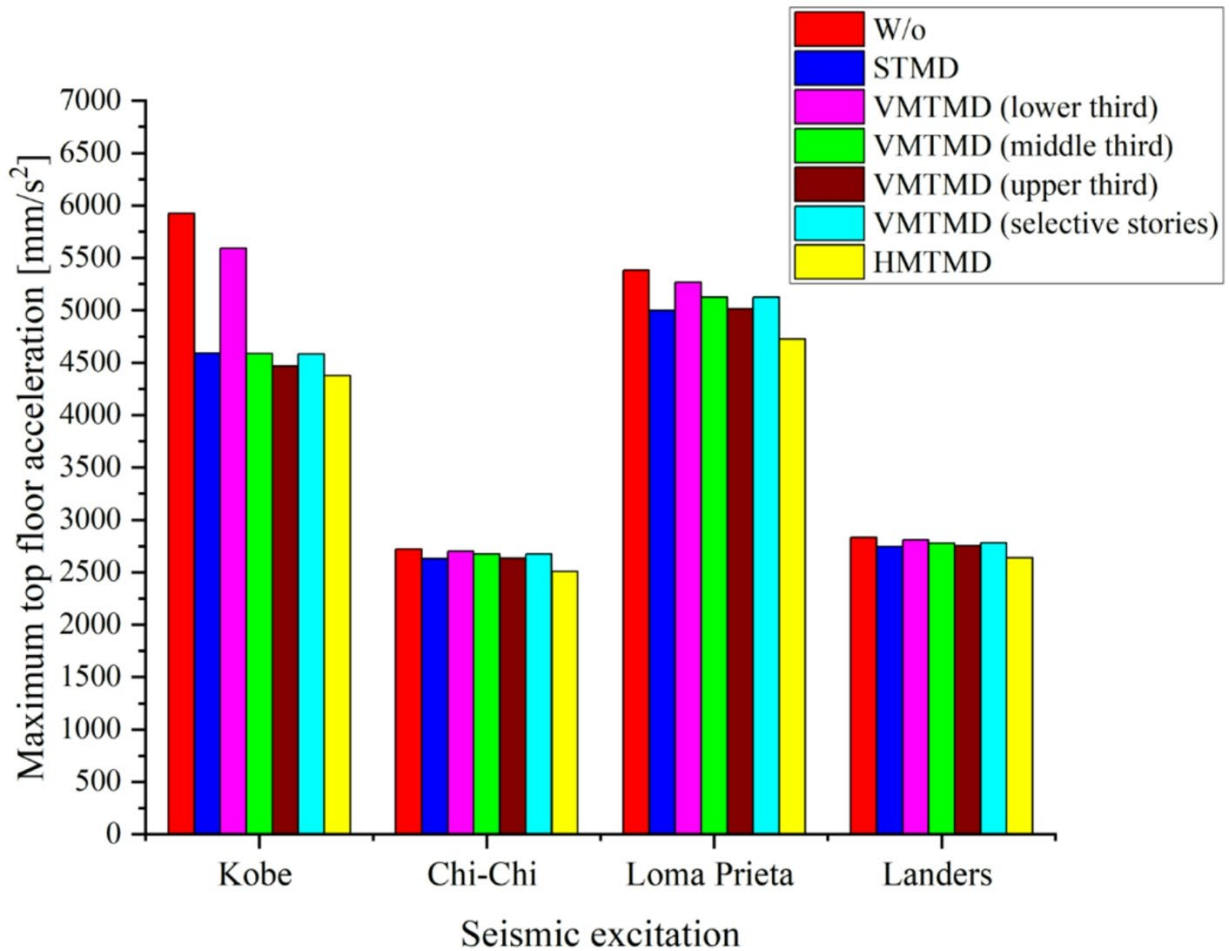
Maximum top-floor acceleration for the investigated TMD layouts under the four scaled earthquake records.

**Fig. 20:**
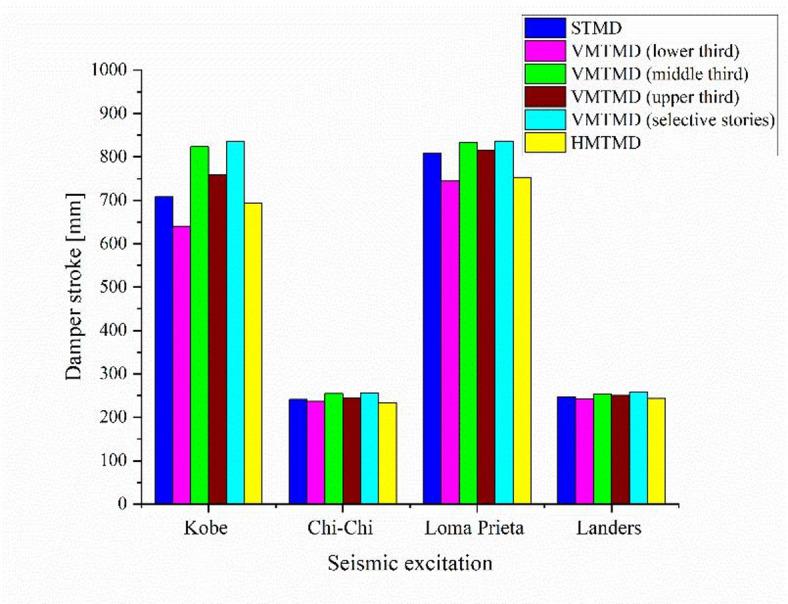
Envelope damper stroke for the controlled configurations across the four scaled earthquake records.

**Fig. 21:**
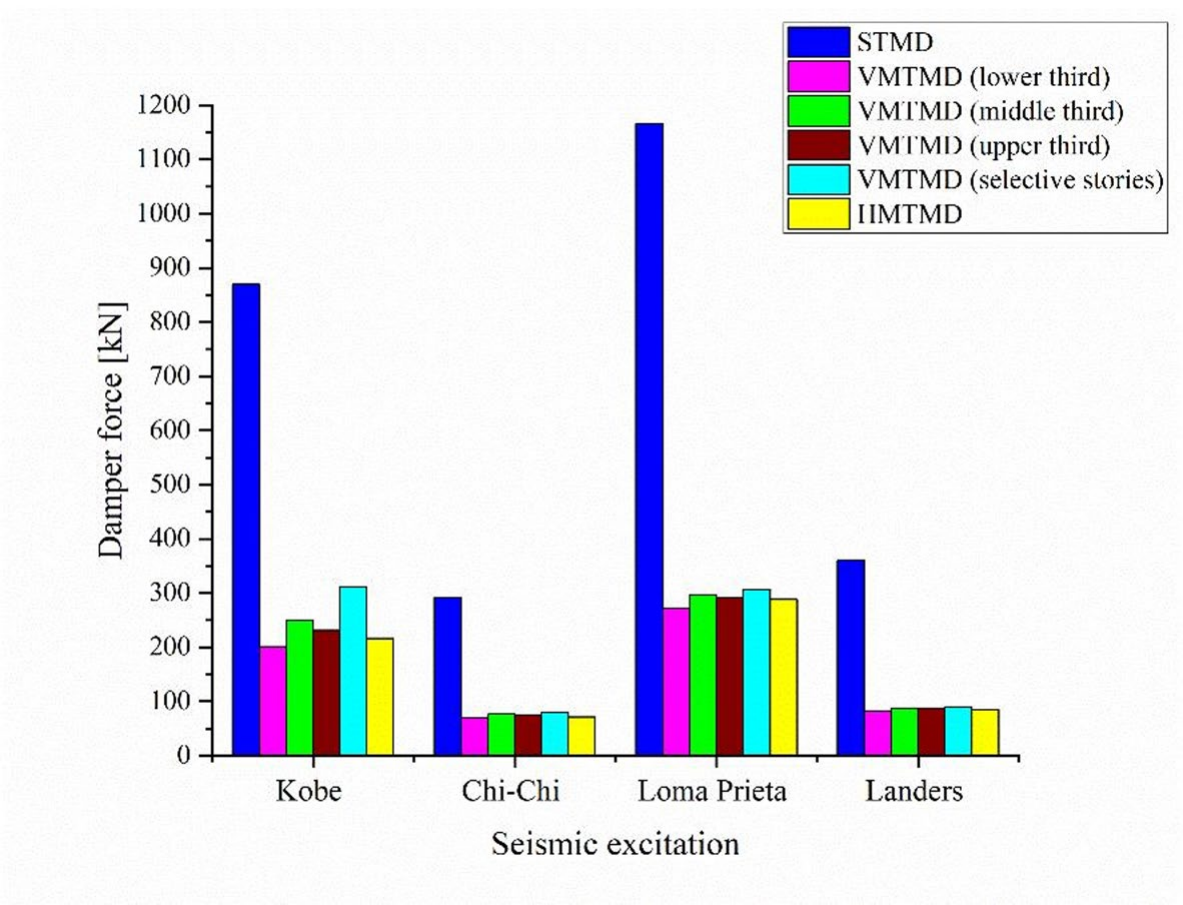
Envelope damper force for the controlled configurations across the four scaled earthquake records.

Figures 17, 18, 19, 20 and 21 provide a graphical summary of selected response quantities reported in Tables 12, 13, 14 and 15, including maximum top-floor displacement, inter-story relative displacement, top-floor acceleration, damper stroke, and damper force.

Tables 12, 13, 14 and 15 show that the response of the regular building varies with both the earthquake record and the adopted TMD layout. The horizontal MTMD arrangement yielded the lowest maximum top-floor displacement among the layouts compared for the four selected records. The same layout also resulted in the lowest inter-story drift displacement in the tabulated cases. The additional force-related quantities provide a broader view of the response than top displacement alone. For Kobe and Chi-Chi excitations, the base shear and overturning moment generally decreased when the auxiliary mass was introduced, with the upper-third VMTMD and HMTMD cases showing comparable reductions in several entries. For Loma Prieta and Landers, the reductions in base shear and overturning moment were smaller than those observed for displacement-based quantities. This difference indicates that a layout that reduces peak displacement does not necessarily reduce all engineering demand parameters by the same proportion. The STMD case generally produced larger damper forces than the distributed MTMD cases in the tabulated records, while the maximum stroke varied with the selected distribution and earthquake input. These values are relevant for checking the practical deformation and force demands of the damper units.

### Detuning sensitivity analysis of horizontally distributed MTMDs

The previous sections compared the response of the regular 12-storey building using STMDs, vertically distributed MTMDs, and horizontally distributed MTMDs under the selected scaled earthquake records. Since the horizontal four-corner MTMD layout produced lower displacement-based response measures than the uncontrolled case and the roof-level STMD in the selected analyses, this configuration was further examined to evaluate its sensitivity to tuning-frequency detuning. The purpose of this section is not to define a universally optimum detuning bandwidth but to assess how small deviations from the target TMD frequency influence the response of the investigated regular-building model.

The detuning ratio was defined relative to the optimum circular frequency obtained from the adopted tuning procedure. Five detuning cases were considered: 0%, ± 2%, ± 4%, ± 6%, and ± 8%. The 0% case represents the reference tuned condition, while the other cases represent symmetric frequency deviations around the target MTMD frequency. For each detuning level, the MTMD stiffness was recalculated using the modified circular frequency, while the assigned MTMD mass was held constant. The comparison was performed using EDPs under the Kobe, Chi-Chi, Loma Prieta, and Landers records. The modified TMD circular frequencies and corresponding stiffness values used in the sensitivity analysis are summarized in Table 16. The EDPs’ response results for the investigated detuning levels are reported in Tables 17, 18, 19 and 20.

**Table 16:** Detuning parameters of the horizontal four-corner MTMD system.

Dampers	Parameters	Tuning condition	Detuning conditions			
		0%	± 2%	± 4%	± 6%	± 8%
TMD 1	${\omega _{TMD}}$(rad/s)	3.213	3.245	3.342	3.309	3.47
	k_TMD_ (N/mm)	295.558	301.475	319.676	313.484	344.73
	c_TMD_ (N.s/mm)	45.405	45.895	`47.222	46.799	49.077
TMD 2	${\omega _{TMD}}$(rad/s)	3.213	3.277	3.084	3.405	3.341
	k_TMD_ (N/mm)	295.558	307.45	272.337	331.937	319.576
	c_TMD_ (N.s/mm)	45.405	46.347	43.585	48.158	47.252
TMD 3	${\omega _{TMD}}$(rad/s)	3.213	3.18	3.149	3.116	2.955
	k_TMD_ (N/mm)	295.558	289.518	283.854	277.982	249.998
	c_TMD_ (N.s/mm)	45.405	44.975	44.497	44.07	41.793
TMD 4	${\omega _{TMD}}$(rad/s)	3.213	3.148	3.277	3.02	3.084
	k_TMD_ (N/mm)	295.558	283.721	307.499	261.117	272.302
	c_TMD_ (N.s/mm)	45.405	44.523	46.313	42.713	43.618

**Table 17:** Detuning sensitivity results under Kobe excitation.

Peak response	Detuning cases				
	0%	± 2%	± 4%	± 6%	± 8%
U_floor_ (mm)	185	188.6	175	180.3	193.1
Drift displacement (mm)	26.4	27.2	25.1	26	27.88
A_floor_ (mm/s^2^)	4376.28	4389.4	4352.1	4364.5	4403.5
Base shear (kN)	107.8	107.95	106.9	107.3	108.2
Over-turning moment (kN · m)	230.8	231.3	228.45	229.6	232.1
Damper stroke (mm)	692.5	699.3	684.6	688.7	704.6
Damper force (kN)	216	216.7	214.5	215.3	217.2

**Table 18:** Detuning sensitivity results under Chi-Chi excitation.

Peak response	Detuning cases				
	0%	± 2%	± 4%	± 6%	± 8%
U_floor_ (mm)	83.5	84.2	81.5	82.45	84.76
Drift displacement (mm)	9.68	9.8	9.35	9.42	9.96
A_floor_ (mm/s^2^)	2507.22	2514.6	2498.6	2502.3	2518.6
Base shear (kN)	57.34	57.8	56.88	57.1	58.02
Over-turning moment (kN · m)	123.78	124.6	122.87	123.24	124.9
Damper stroke (mm)	233.45	238.4	226.3	229.6	241.34
Damper force (kN)	71.24	71.81	70.45	70.86	72.01

**Table 19:** Detuning sensitivity results under Loma Prieta excitation.

Peak response	Detuning cases				
	0%	± 2%	± 4%	± 6%	± 8%
U_floor_ (mm)	235	232.3	228	230.8	237.8
Drift displacement (mm)	27.8	27.23	26.1	26.85	28.14
A_floor_ (mm/s^2^)	4725.73	4705.63	4683.5	4695.2	4736.4
Base shear (kN)	157.758	157.12	156.34	156.87	158.24
Over-turning moment (kN · m)	339.02	338.24	337.3	337.74	339.58
Damper stroke (mm)	752.46	747.3	741.2	745.1	756.7
Damper force (kN)	288.372	287.64	286.56	287.05	288.78

**Table 20:** Detuning sensitivity results under Landers excitation.

Peak response	Detuning cases				
	0%	± 2%	± 4%	± 6%	± 8%
U_floor_ (mm)	96.5	95.2	94	95.6	96.9
Drift displacement (mm)	11.52	11.1	10.8	11.34	11.83
A_floor_ (mm/s^2^)	2639.4	2634.5	2628.4	2638.4	2646.6
Base shear (kN)	68.23	67.8	67.2	68.03	68.48
Over-turning moment (kN · m)	146.74	145.8	145.35	146.27	147.16
Damper stroke (mm)	243.6	236.4	230.7	239.65	247.6
Damper force (kN)	85.34	84.87	84.13	84.74	85.75

**Fig. 22:**
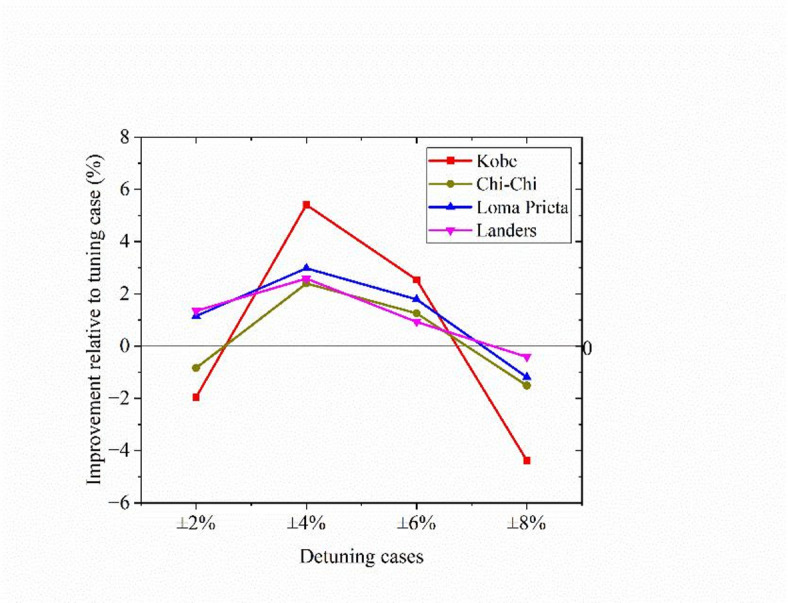
Percentage improvement in maximum top lateral displacement relative to the 0% detuning case for the horizontal four-corner MTMD layout.

**Fig. 23:**
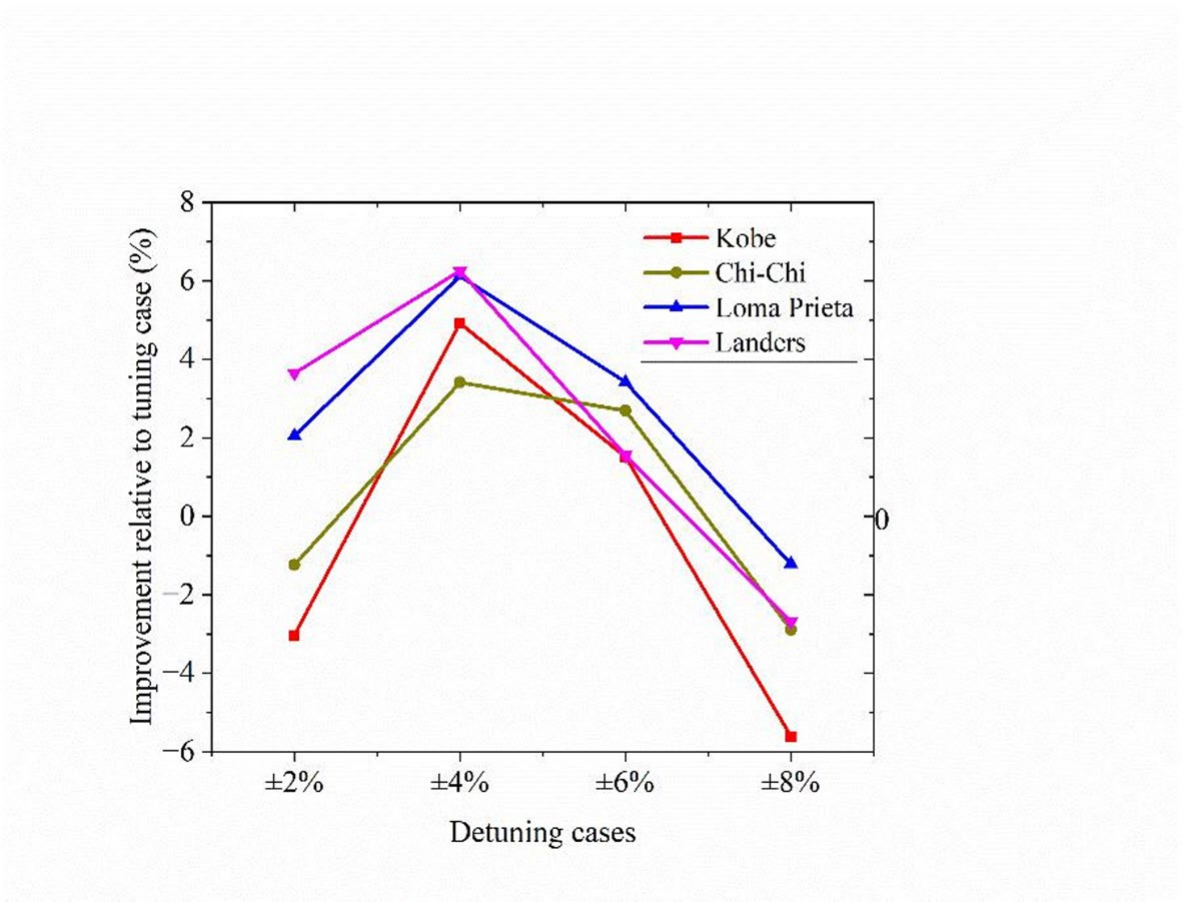
Percentage improvement in maximum inter-story displacement relative to the 0% detuning case for the horizontal four-corner MTMD layout.

**Fig. 24:**
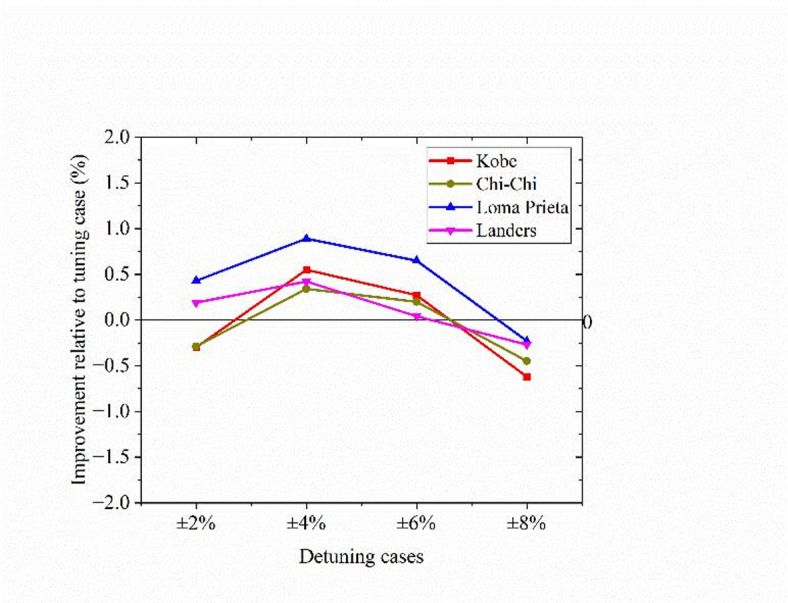
Percentage improvement in maximum top floor acceleration relative to the 0% detuning case for the horizontal four-corner MTMD layout.

**Fig. 25:**
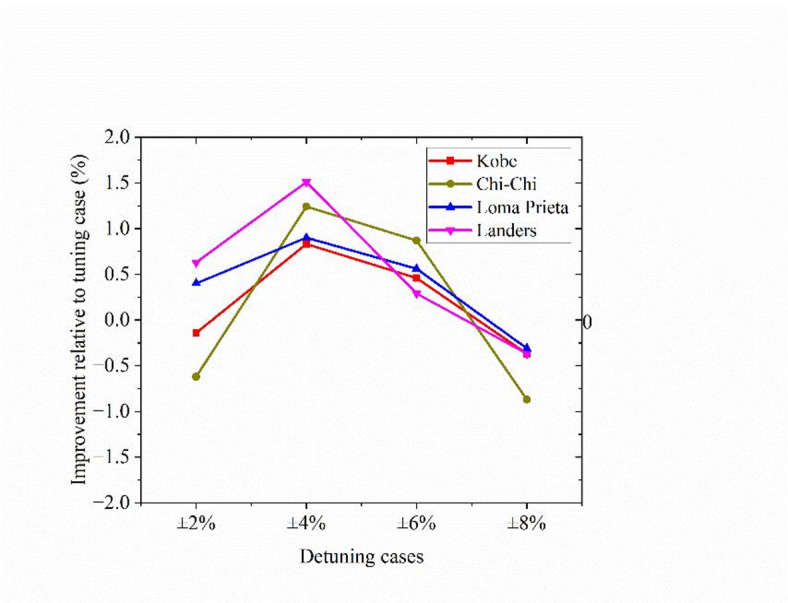
Percentage improvement in maximum base shear relative to the 0% detuning case for the horizontal four-corner MTMD layout.

**Fig. 26:**
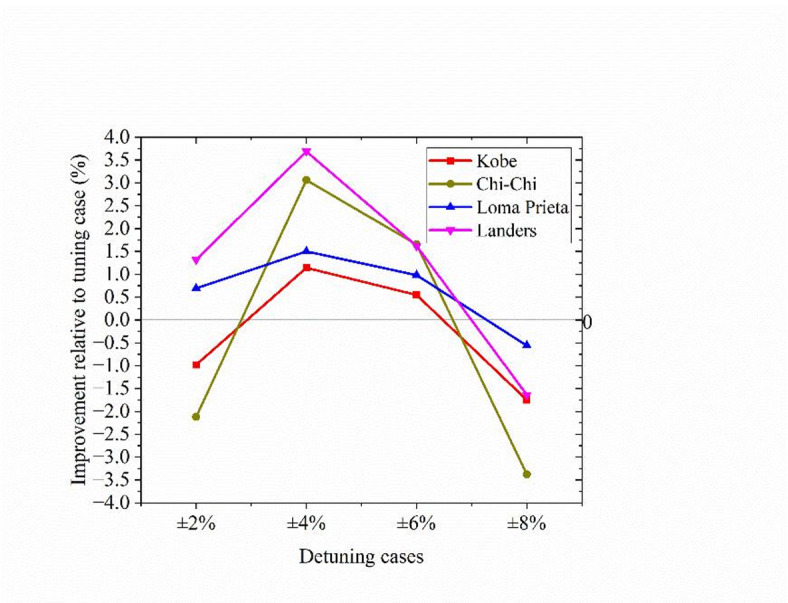
Percentage improvement in maximum damper stroke relative to the 0% detuning case for the horizontal four-corner MTMD layout.

**Fig. 27:**
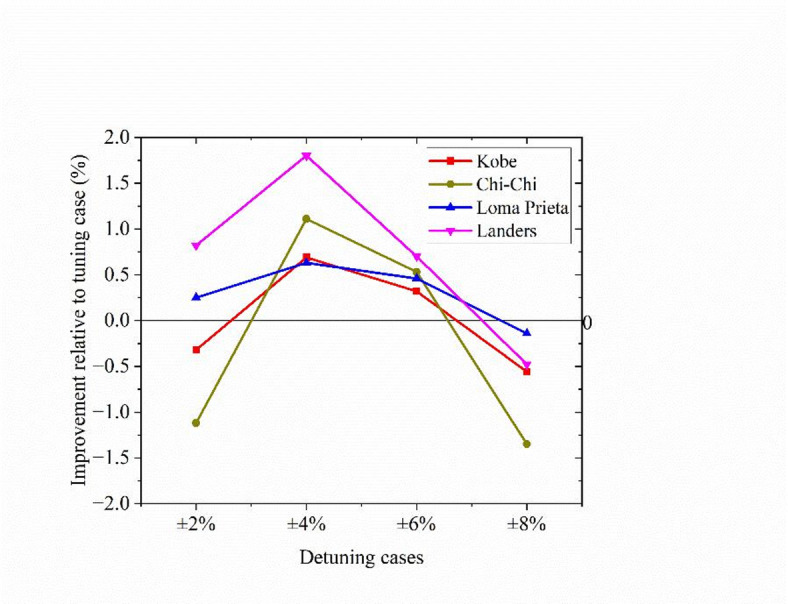
Percentage improvement in maximum damper force relative to the 0% detuning case for the horizontal four-corner MTMD layout.

To visualize the detuning trend, the percentage changes in EDPs are plotted in Figs. 22, 23, 24, 25, 26 and 27, respectively. These figures use the 0% detuning case as the reference response for each earthquake record; therefore, positive values indicate a reduction relative to the reference tuned case.

The percentage change results show that the response of the horizontal four-corner MTMD layout depends on the selected detuning level and the earthquake record. Based on the percentage changes calculated from Tables 17, 18, 19 and 20, the ± 4% case generally produced the largest reduction relative to the 0% detuning case. This trend is most clearly observed for maximum top lateral displacement and maximum inter-story relative displacement, as shown in Figs. 22 and 23, where the ± 4% case gave the lowest response for all four selected records. For maximum top lateral displacement, the improvements at ± 4% relative to the 0% case were 5.41%, 2.40%, 2.98%, and 2.59% under Kobe, Chi-Chi, Loma Prieta, and Landers excitation, respectively. For maximum inter-story relative displacement, the corresponding improvements were 4.92%, 3.41%, 6.12%, and 6.25%.

These reductions are moderate and record-dependent, but they indicate that the ± 4% detuning case was the most favorable among the investigated detuning levels for the selected regular-building model and MTMD parameters. The results also show that the detuning response is not strictly monotonic. For example, the ± 2% case didn’t always improve the response relative to the 0% case, while larger detuning levels such as ± 6% and ± 8% did not provide additional benefit in most response quantities. This behavior suggests that excessive deviation from the target tuning frequency may reduce the effectiveness of the MTMD units for the investigated cases. Therefore, the ± 4% result should be interpreted as a case-specific sensitivity finding rather than as a universal optimum detuning bandwidth. The additional response quantities provide useful context for interpreting the displacement results. The ± 4% case also tended to reduce base shear, overturning moment, damper stroke, and damper force relative to the 0% case, although the magnitude of these reductions was smaller for some quantities than for displacement-based measures. Floor acceleration showed only limited changes across the detuning levels.

**Fig. 28:**
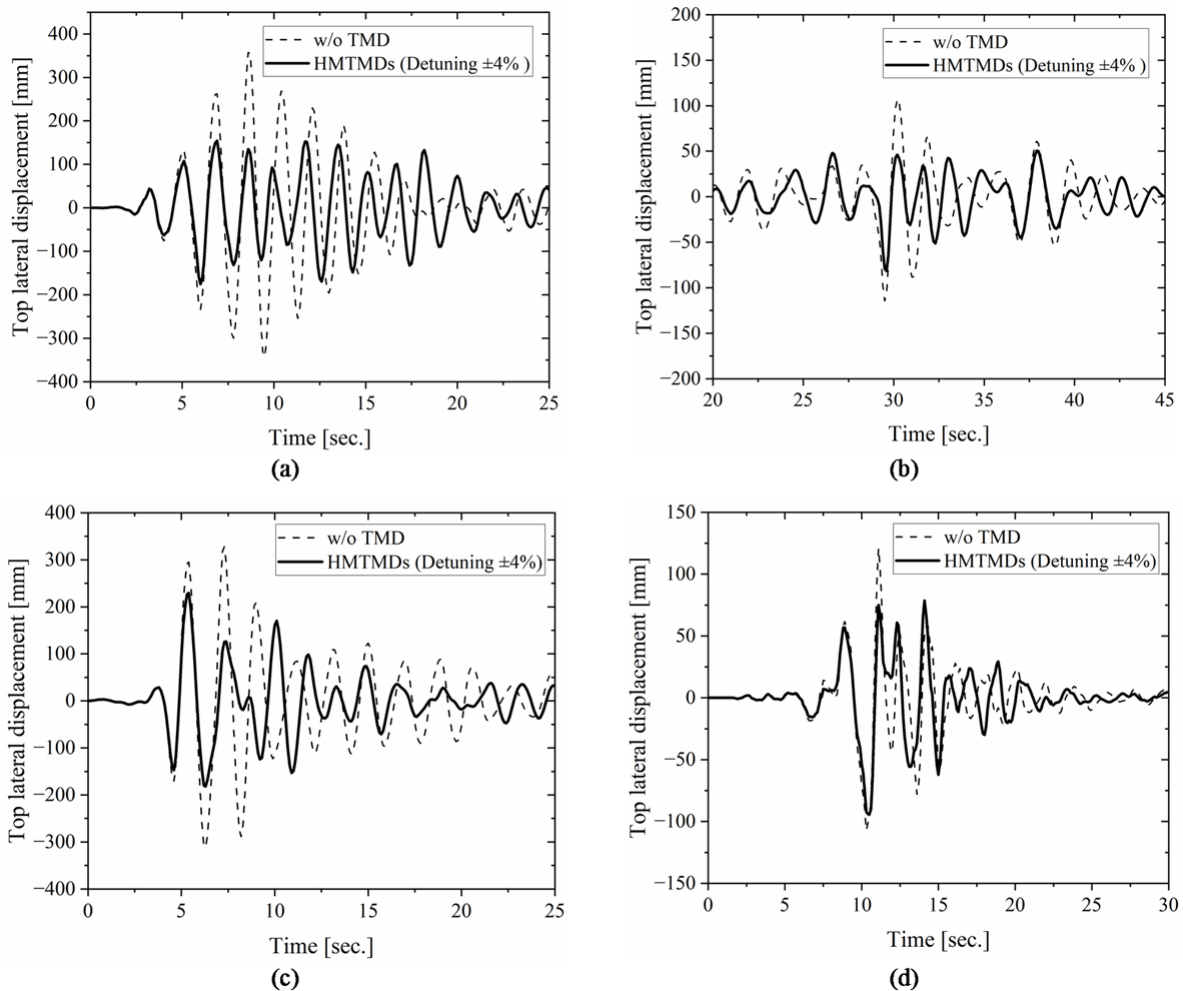
Top lateral displacement of the building with and without HMTMDs on the top floor, with slight detuning ±4% under (a) Kobe earthquake excitation, (b) Chi-Chi earthquake excitation, (c) Loma Prieta earthquake excitation, and (d) Landers earthquake excitation.

**Fig. 29:**
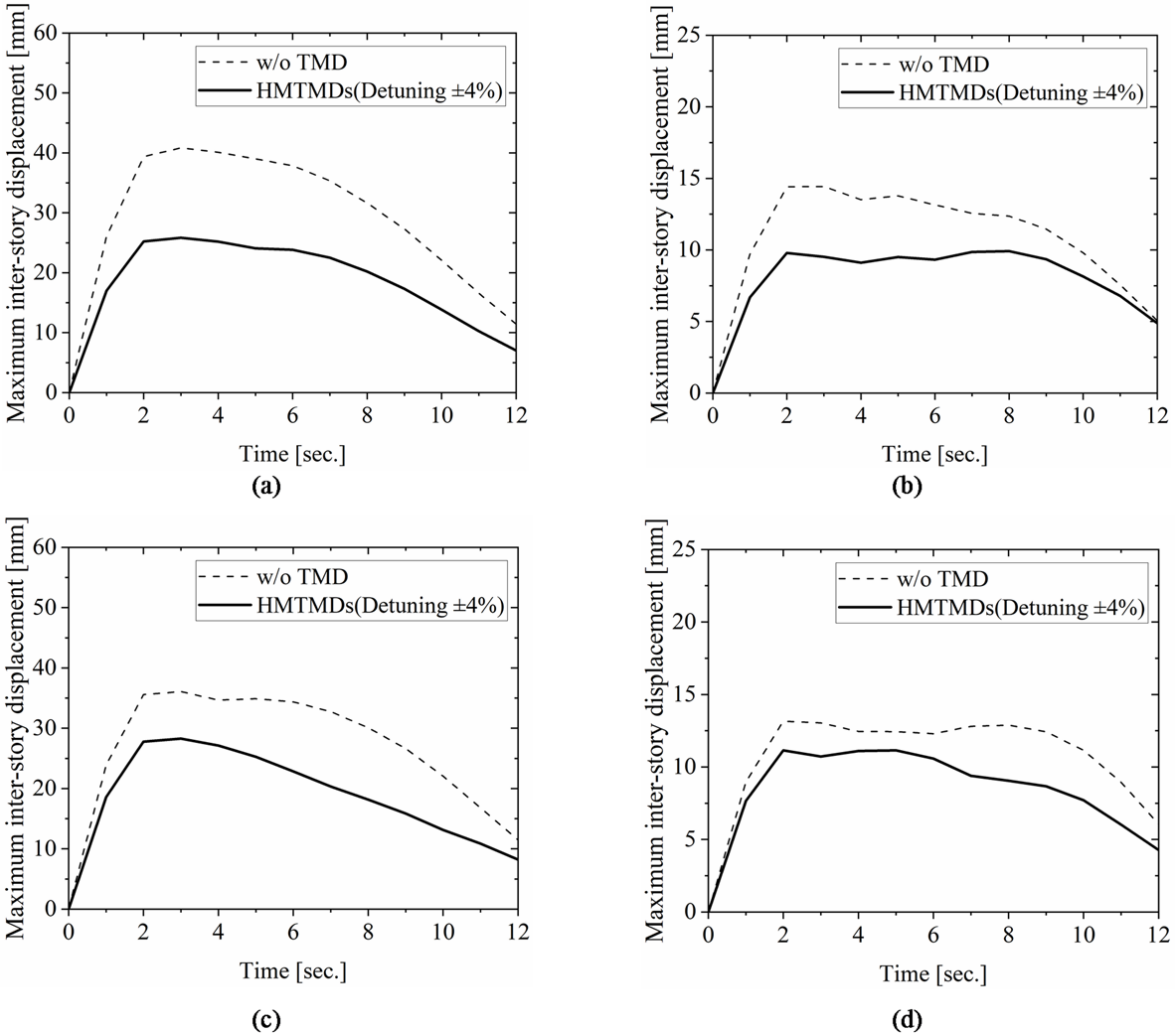
Maximum relative displacement between stories of the building with and without HMTMDs on the top floor, with detuning ± 4% under (a) Kobe earthquake excitation, (b) Chi-Chi earthquake excitation, (c) Loma Prieta earthquake excitation, and (d) Landers earthquake excitation.

These observations indicate that the detuning comparison should be evaluated using multiple response measures, rather than relying only on one peak displacement value. Based on these results, the ± 4% detuning case was adopted as the representative detuned MTMD configuration for the response comparison with the uncontrolled case for the time-history responses of top lateral displacement and inter-story relative displacement, as shown in Figs. 28 and 29. However, the present analysis remains limited to the selected regular 12-story building, the adopted damper properties, and the four scaled earthquake records.

**Fig. 30:**
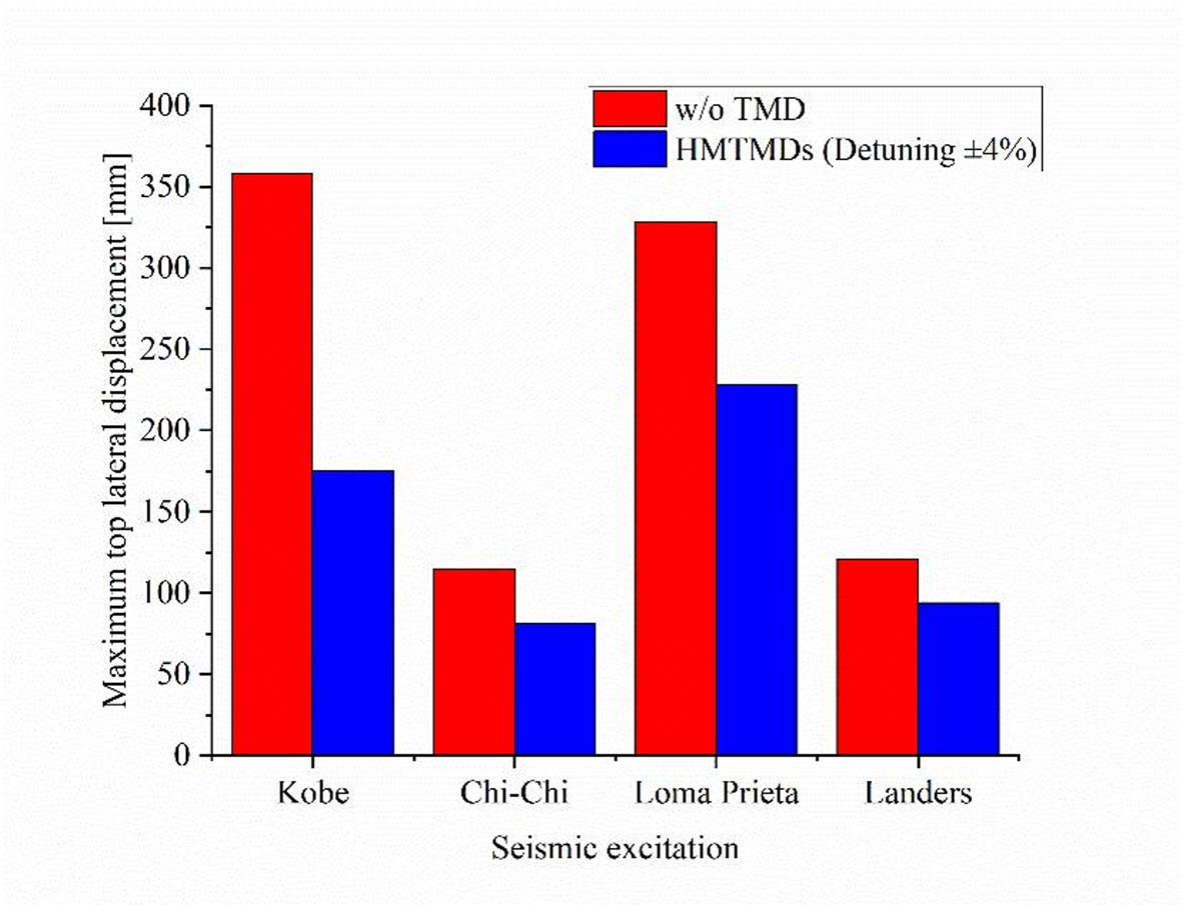
Maximum top lateral displacement of the model under seismic records with and without HMTMDs with detuning ± 4% on the top floor.

The results show that the ± 4% case produced the lowest maximum top lateral displacement and inter-story relative displacement among the investigated detuning levels for the four selected records. Relative to the uncontrolled building, the ± 4% case reduced maximum inter-story relative displacement by 36.7%, 32.15%, 21.6%, and 15.3%, and maximum top lateral displacement by 51.13%, 29.13%, 30.5%, and 22.3% under the Kobe, Chi-Chi, Loma Prieta, and Landers records, as shown in Figs. 29 and 30, respectively. Therefore, ± 4% is treated in this study as the most favorable sensitivity case for the selected model and records, rather than as a universally optimized detuning bandwidth.

## Numerical analysis of MTMDs in the irregular models

**Fig. 31:**
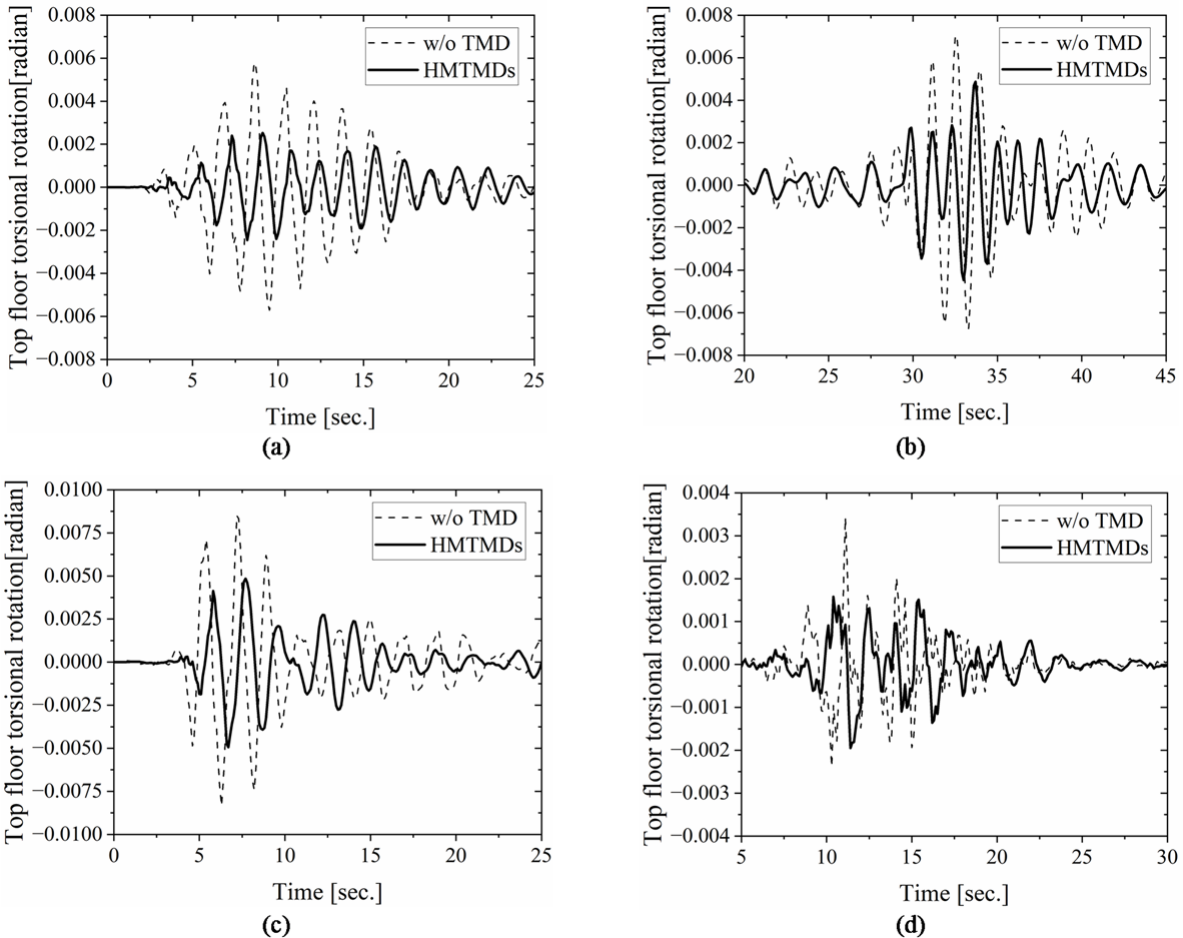
Top-floor torsional rotation of the T-shaped building with and without HMTMDs on the top floor under (a) Kobe, (b) Chi-Chi, (c) Loma Prieta, and (d) Landers earthquake excitation.

**Fig. 32:**
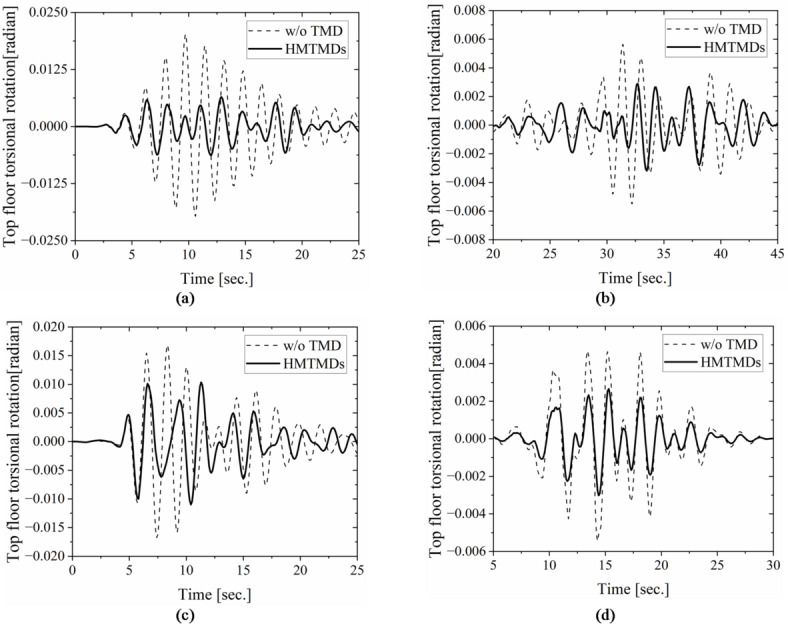
Top-floor torsional rotation of the C-shaped building with and without HMTMDs on the top floor under (a) Kobe, (b) Chi-Chi, (c) Loma Prieta, and (d) Landers earthquake excitation.

**Fig. 33:**
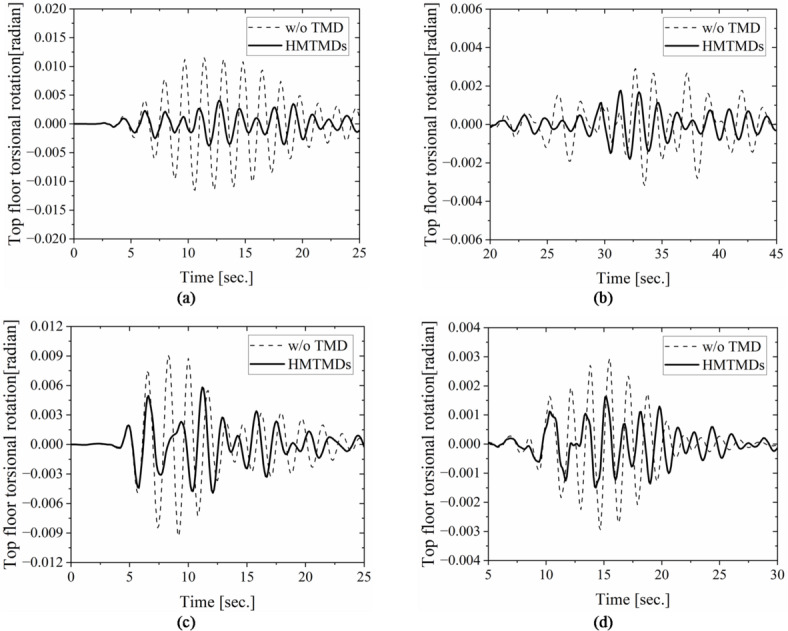
Top-floor torsional rotation of the L-shaped building with and without HMTMDs on the top floor under (a) Kobe, (b) Chi-Chi, (c) Loma Prieta, and (d) Landers earthquake excitation.

**Fig. 34:**
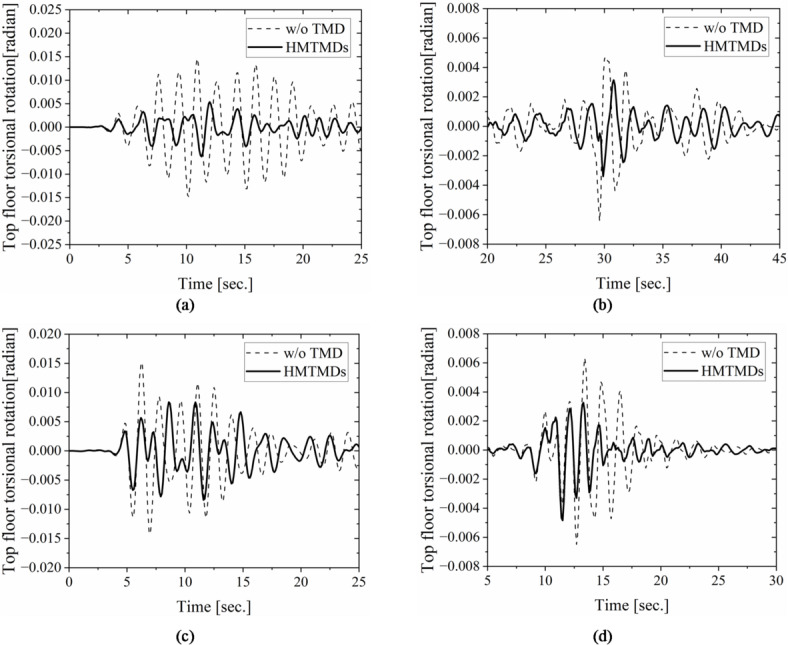
Top-floor torsional rotation of the Stadium-shaped building with and without HMTMDs on the top floor under (a) Kobe, (b) Chi-Chi, (c) Loma Prieta, and (d) Landers earthquake excitation.

For the irregular plan configurations (T, C, L, and stadium), the perimeter-based HMTMD arrangement was examined because CM–CR eccentricity can contribute to coupled lateral–torsional response. The damper locations were selected at boundary regions to provide relatively large torsional lever arms with respect to the CR, as described in sections  “Dynamic eccentricity and MTMD location coordinates. The Kobe, Chi-Chi, Loma Prieta, and Landers records were used to evaluate the response of this layout in terms of top-floor torsional rotation, as shown in Figs. 31, 32, 33 and 34.

In addition to the time-history comparison of top torsional rotation, the irregular-building response was further assessed using selected EDPs. To provide a concise comparison across the investigated plan configurations, the percentage reduction achieved by the perimeter-based MTMD layout was calculated for the maximum top-floor displacement, maximum top-floor torsional rotation, base shear, and maximum top-floor acceleration under the selected scaled earthquake records. These results are summarized in Table 21.

**Table 21:** Reduction improvement of selected response quantities for the irregular buildings.

Earthquake	Shapes	Reduction improvement %			
		Maximum top- floor displacement (U)	Maximum top- floor torsional rotation ($\theta )$	Base shear (V)	Maximum top- floor acceleration (A)
Kobe	T	52.3	64.4	31.3	34.7
	C	50.8	68	28	31.6
	L	46.6	64.8	26.5	28.3
	Stadium	50.5	57.7	29.2	32.2
Chi-Chi	T	28.3	46.5	20.7	14.6
	C	23.6	43.5	17.3	16.5
	L	32.5	41.3	24.6	20.3
	Stadium	24.3	31.2	22.3	18.6
Loma Prieta	T	18.5	41.75	23.5	16.8
	C	34.22	38.1	28.6	20.7
	L	24.5	47.2	26.1	19.5
	Stadium	19.4	44.3	24.8	15.3
Landers	T	20.5	40.8	17.3	12.2
	C	18.3	44.5	15.2	14.8
	L	23.6	45.7	18.7	17.1
	Stadium	21.2	25.4	15.3	13.6

**Fig. 35:**
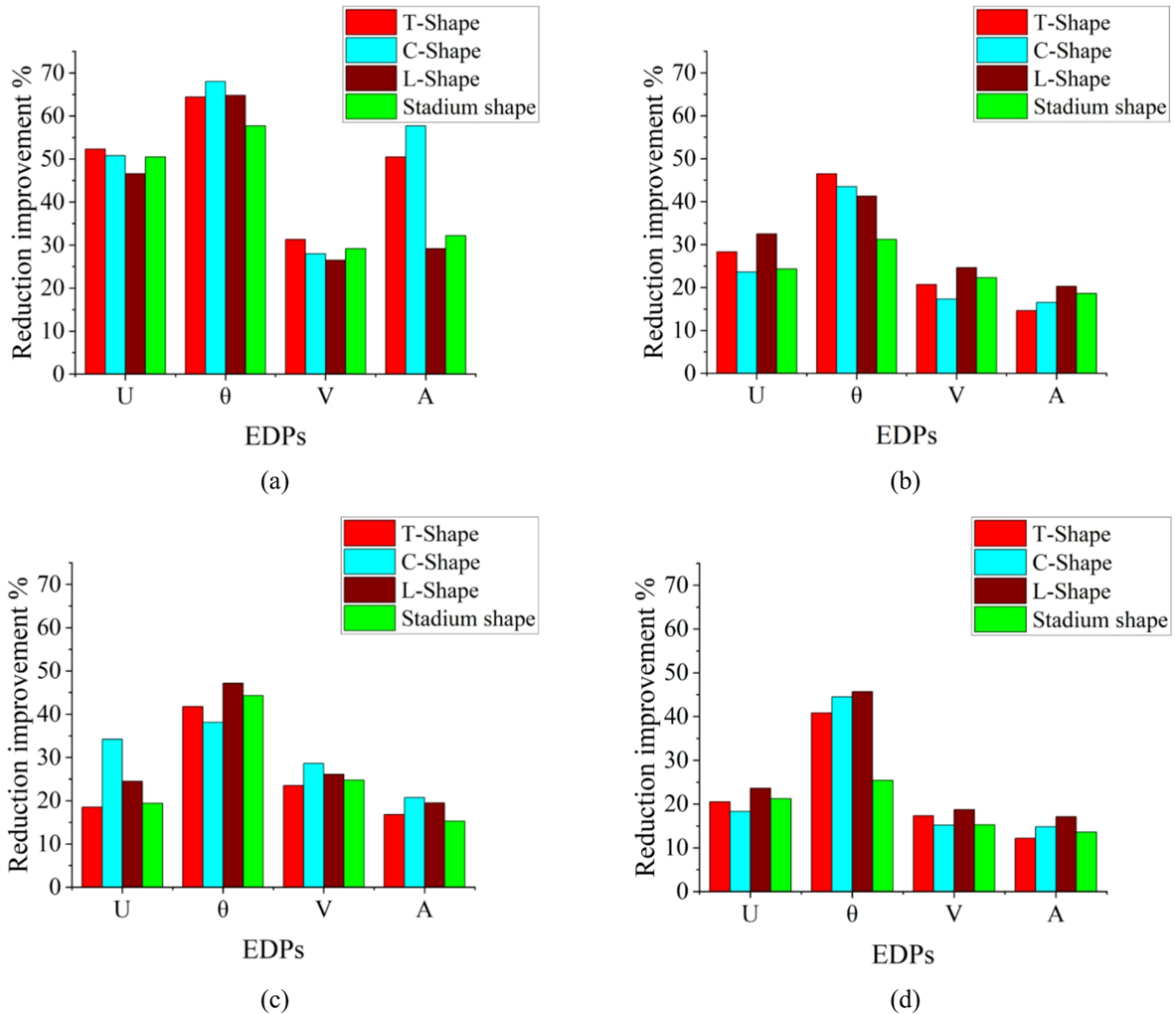
Percentage reduction in selected EDPs of the irregular buildings with perimeter-based MTMDs under (a) Kobe, (b) Chi-Chi, (c) Loma Prieta, and (d) Landers earthquake excitation.

To facilitate comparison among the investigated irregular configurations and earthquake records, the percentage reductions listed in Table 21 are further presented in chart form in Fig. 35. The chart is intended to highlight the relative variation of the selected response quantities among the T-, C-, L-, and stadium-shaped buildings.

As shown in Table 21; Fig. 35, the percentage reductions varied with both the building shape and the selected response quantity. These results indicate that the effectiveness of MTMDs in irregular buildings depends not only on the tuning frequency and mass ratio but also on the spatial relationship between damper locations, the CM, and the CR. In asymmetric plans, the CM–CR offset produces a coupled lateral–torsional response; therefore, a roof-centered or centrally placed damper may have limited torsional leverage even if it is effective in reducing translational displacement. By distributing the same total auxiliary mass along the perimeter, the proposed arrangement increases the torsional moment arms of the MTMD units relative to the CR, thereby allowing the dampers to contribute more directly to controlling the coupled response. This provides a possible explanation for the observed reduction in torsional rotation in the investigated irregular models.

The comparison with other control technologies should be interpreted within the scope of the present study. Seismic isolation can substantially reduce the seismic input transmitted to the superstructure and may also reduce torsional coupling by adjusting the stiffness and horizontal distribution of isolation bearings. Similarly, negative-stiffness dampers, tuned viscous mass dampers, and inerter-based systems may provide high control efficiency through enhanced energy dissipation or apparent mass-amplification mechanisms. However, the objective of this work is not to establish the superiority of MTMDs over these systems. Rather, the study evaluates whether an equivalent auxiliary mass, selected within a practical passive-control range, can be used more efficiently by changing its spatial distribution. From this perspective, the proposed MTMD strategy represents a floor- or roof-level passive control option that may be relevant when foundation-level isolation or specialized mechanical devices are not adopted.

A direct comparative study between perimeter-based MTMDs, base-isolated systems with optimized bearing stiffness distribution, and inerter- or negative-stiffness-based dampers remains an important direction for future work. Such a comparison would help identify the conditions under which each control strategy is preferable in terms of lateral response reduction, torsional control, constructability, sensitivity to detuning, and retrofit feasibility.

## Conclusion

This study examined the seismic response of regular and irregular 12-story steel buildings equipped with single and multiple tuned mass dampers using nonlinear time-history analysis in ABAQUS. The investigated configurations included a roof-level STMD, vertically distributed MTMDs, horizontally distributed MTMDs, slightly detuned MTMDs, and perimeter-based MTMD arrangements for irregular plan configurations. The main conclusions are as follows:

1. For the regular building, increasing the STMD mass ratio improved the reduction in maximum top lateral displacement for the selected records. However, concentrating a large auxiliary mass at a single location may require additional checks related to load transfer and installation; these practical aspects were not quantified in the present study.
2. Among the investigated vertical MTMD arrangements, placing the dampers in the upper third of the building produced the largest reduction in top lateral displacement, with reductions of 45.3%, 18.26%, 20.3%, and 14.5% under the Kobe, Chi-Chi, Loma Prieta, and Landers records, respectively.
3. For the regular building, the horizontal four-corner MTMD arrangement produced lower maximum top lateral displacement than the roof-level STMD in the selected analyses. Compared with the STMD case, the tuned horizontal MTMD layout reduced the maximum top lateral displacement by 2.23%, 5.66%, 7.35%, and 5.37% under the Kobe, Chi-Chi, Loma Prieta, and Landers records, respectively. When tuning-frequency deviations of 0%, ± 2%, ± 4%, ± 6%, and ± 8% were considered, the ± 4% case produced the lowest maximum top lateral displacement and inter-story relative displacement for all four selected records. Relative to the uncontrolled building, the ± 4% case reduced the maximum top lateral displacement by 51.13%, 29.13%, 30.5%, and 22.3%, and reduced the inter-story relative displacement by 36.7%, 32.15%, 21.6%, and 15.3% under the Kobe, Chi-Chi, Loma Prieta, and Landers records, respectively. Therefore, the ± 4% detuning level is interpreted as the most favorable case among the investigated detuning levels for the selected model and records, rather than as a universally optimized detuning bandwidth.
4. For the irregular buildings, the perimeter-based MTMD arrangement was evaluated with respect to CM–CR eccentricity and the torsional lever arms of the damper units. The assessment was extended to four scaled earthquake records and included maximum top-floor displacement, maximum top-floor torsional rotation, base shear, and maximum top-floor acceleration. Across the investigated T-, C-, L-, and stadium-shaped models, the reduction in maximum top-floor displacement ranged from 18.3% to 52.3%, while the reduction in maximum top-floor torsional rotation ranged from 25.4% to 68.0%. The corresponding reductions in base shear and maximum top-floor acceleration ranged from 15.2% to 31.3% and from 12.2% to 34.7%, respectively. These results indicate that the perimeter-based MTMD layout reduced the selected response quantities for the investigated irregular models and records.
5. The irregular building results indicate that MTMD effectiveness is influenced not only by mass ratio and tuning frequency but also by the spatial relationship between damper locations, CM, CR, and the resulting torsional lever arms. Placing the MTMD units along the perimeter allows the same auxiliary mass to participate more directly in reducing coupled lateral–torsional response. The reductions reported in displacement, torsional rotation, base shear, and top-floor acceleration support the relevance of the CM–CR/eccentricity-based placement strategy for the investigated irregular models.
6. Other control technologies, including seismic isolation, negative-stiffness dampers, tuned viscous mass dampers, and inerter-based systems, may also provide effective seismic response reduction. The present study is limited to MTMDs as a passive floor- or roof-level control strategy and does not compare them numerically with these alternatives. Future work should compare perimeter-based MTMDs with base-isolated systems and inerter- or negative-stiffness-based dampers under the same ground-motion suite.

The conclusions are limited to the selected structural models, TMD parameters, engineering demand parameters, ground-motion records, and scaling procedure adopted in this study. Future studies should include larger ground motions with multiple intensity levels, damper-failure cases, cost-constructability assessments, and frequency-domain or energy-based verification.

## Data Availability

The data and materials supporting the findings of this study are available from the authors upon reasonable request.
